# Supplementary material for: Site-selected in situ polymerization for living cell surface engineering
Source: Nat Commun. 2023 Nov 10;14:7285. doi: 10.1038/s41467-023-43161-x (PMC10638357; doi:10.1038/s41467-023-43161-x)
Supplement: Supplementary file 1 — Supplementary Information [file 41467_2023_43161_MOESM1_ESM.pdf]

Supplementary Information

Site-Selected *in situ* Polymerization for Living Cell Surface Engineering

Yihong Zhong,[1] Lijia Xu,[1] Chen Yang,[1] Le Xu,[2] Guyu Wang,[1] Yuna Guo,[1] Songtao Cheng,[1] Xiao Tian,[3] Changjiang Wang,[3] Ran Xie,[3,4] Xiaojian Wang,*,[2] Lin Ding,*,[1,4] Huangxian Ju[1]

[1]State Key Laboratory of Analytical Chemistry for Life Science, School of Chemistry and Chemical Engineering, Nanjing University, Nanjing 210023, China.

[2]Institute of Advanced Synthesis, School of Chemistry and Molecular Engineering, Nanjing Tech University, Nanjing 211816, China.

[3]State Key Laboratory of Coordination Chemistry, School of Chemistry and Chemical Engineering, Nanjing University, Nanjing 210023, China.

[4]Chemistry and Biomedicine Innovation Center (ChemBIC), Nanjing University, Nanjing 210023, China.

*Corresponding Authors:

Lin Ding (dinglin@nju.edu.cn)

Xiaojian Wang (ias_xjwang@njtech.edu.cn)

Table of Contents

1. Supplementary Methods

1.1 Materials and Reagents.

1.2 Apparatus.

1.3 Cell Culture.

1.4 Sample preparation procedure for CLSM and STED imaging.

1.5 Sample preparation and analysis for FCM.

1.6 Synthesis.

- Synthesis of chain transfer agent (CTA) 2-{[(butylsulfanyl)carbonothioyl]sulfanyl}propanoic acid (BTPA).
- Synthesis of DBCO-BTPA.
- Synthesis of DBCO-SS-BTPA.
- Synthesis of N-(2-hydroxypropyl) methacrylamide (HPMA).
- Synthesis of methacrylamide-mannose (MA-Man) glycomonomer.
- Synthesis of methacrylamide-galactose (MA-Gal) glycomonomer.

1.7 CCK-8 assay of the cytotoxic effect of polymerization components.

1.8 Solution Fenton-RAFT polymerization.

1.9 Cell viability assay.

1.10 Cell proliferation assay.

1.11 Measurement of the number of azides on the cell surface using ^{19}F NMR

1.12 SEM imaging of Cell-P.

1.13 Intracellular reactive oxygen species assay.

2. Supplementary Figures

3. Supplementary References

1. Supplementary Methods

1.1 Materials and Reagents.

With the exceptions noted below, all commercially available reagents and laboratory supplies were purchased from Sigma-Aldrich Co. (USA). All lectins used in this article were obtained from Vector Labs (USA). N-azidoacetylgalactosamine-tetraacylated ($Ac_4GalNAz$) and 1-azidoethyl-choline (AECho) were obtained from Prof. Ran Xie's lab at Nanjing University. Hydrogen peroxide (H_2O_2, 35% w/w) was purchased from Alfa Aesar (USA). Dibenzoazacyclooctyne-disulfide-amine (DBCO-SS-NH_2, purity 95%) and DBCO-poly(ethylene glycol)$_3$-F (DBCO-PEG$_3$-F, purity 95%) were purchased from Xi'an confluore Biotech Co., Ltd. (China). Acrylamide-PEG$_4$-azide (AA-PEG$_4$-azide, purity 95%) and AA-PEG$_4$-biotin (purity 95%) were purchased from Xi'an Ruixi Biotech Co., Ltd. (China). Neuraminidase (NEU, from *Clostridium perfringens*) was purchased from Aladdin Biotech Co., Ltd. (China). Phalloidin-iFluor 594 and goat anti-rabbit IgG H&L (Alexa Fluor® 488, pre-adsorbed) were purchased from Abcam (UK). FITC-conjugated anti-human CD3 antibody (Clone HIT3a) and PE-conjugated anti-human CD69 antibody (Clone FN50) were purchased from BioLegend, Inc. (USA). RIPA lysis buffer I, phenylmethylsulfonyl fluoride, 20× Tris buffered saline (TBS) (pH 7.4), polyvinylidene fluoride (PVDF) membrane, paraformaldehyde (4%) and galactose oxidase (GAO) were obtained from Shanghai Sangon Biotech Co., Ltd. (China). HRP-labeled goat anti-rabbit IgG (H+L) and protease inhibitor (50×) were purchased from Beyotime Biotech Co., Ltd. (China). 12% TGX Stain-Free™ FastCast™ kit was from Bio-Rad Inc. (USA). Seahorse XF Cell Mito Stress Test Kit and XF RPMI base medium were purchased from Agilent Tech. Co. Ltd. (USA). Phospho-histone H2A.X (Ser139) (20E3) rabbit mAb, caspase-3 (D3R6Y) rabbit mAb, caspase-9 antibody and cytochrome c antibody were purchased form Cell Signaling Technology, Inc. (USA). Roswell Park Memorial Institute 1640 medium (RPMI-1640, containing 2.0 mg/mL D-glucose, 0.3 mg/mL glutamine, 2.0 mg/mL $NaHCO_3$, 80 U/mL penicillin, and 0.08 mg/mL streptomycin), phosphate buffered saline (PBS, containing 136.7 mM NaCl, 2.7 mM KCl, 8.72 mM Na_2HPO_4, 1.41 mM KH_2PO_4), annexin V-FITC/propidium iodide (PI) apoptosis assay kit, cell counting kit-8 (CCK-8), Triton X-100 were purchased from KeyGEN Biotech Co., Ltd. (China). Methionine-free RPMI-1640 was customized from Boster Biotech Co., Ltd. (China). Phenol red-free RPMI-1640 was purchased from SenBeiJia Biotech Co., Ltd. (China). PageRuler™ prestained protein ladder (10 to 180 kDa), Pierce™ BCA protein assay kit, fetal bovine serum (FBS), GlutaMAX™ supplement, sodium pyruvate, B-27 supplement minus AO (50×) and LIVE/DEAD™ Fixable Far-Red Dead Cell Stain Kit were purchased from Thermo Fisher Scientific Inc. (USA). All aqueous solutions were prepared with ultrapure water (≥18 MΩ, Milli-Q, Millipore).

1.2 Apparatus.

Fluorescence imaging of cells were performed on an SP8 stimulated emission depletion (STED) 3X confocal laser scanning microscopy (CLSM, Leica, Germany) and images were analyzed with Leica Application Suite X (LAS X, version 3.3.0) or ImageJ (version 1.53c) software. Flow cytometric (FCM) analysis was performed on a CytoFLEX flow cytometer (Beckman-Coulter, USA) using FlowJo (version 10.5.3) software for data analysis. Cell number was calculated using a Countess® II FL Automated Cell Counter (Thermo Fisher Scientific, USA). The CCK-8 and BCA measurements were performed on a Varioskan Flash spectral scanning multi-mode reader (Thermo Fisher Scientific, USA), and the data were processed in Microsoft Excel software 2016. Western blots were visualized with a ChemiDoc XRS system (Bio-Rad, USA). Sodium dodecyl sulfate-polyacrylamide gel electrophoresis (SDS-PAGE) was performed on an Electrophoresis Analyzer (Bio-Rad, USA). Cellular metabolism was measured with the Seahorse XFe96 Analyzer (Agilent Technologies, USA) and data were analyzed with Wave (version 2.6.1) software. Gel permeation chromatography (GPC) analysis was performed on an Agilent GPC 1260 Infinity II using N, N-dimethylformamide (DMF) as the eluent at 1 mL/min at 50 °C. The GPC was calibrated with PMMA standards. ^{1}H nuclear magnetic resonance (NMR), ^{13}C NMR, and ^{19}F NMR were recorded on an Avance III 400 spectrometer (Bruck, Germany) at 298 K (^{1}H NMR at 400 MHz, ^{13}C NMR at 100 MHz, and ^{19}F NMR at 376 MHz). For organic small molecules, deuterated solvent was used as reference and sample concentration was approximately 10 $mg \cdot mL^{-1}$. All NMR data were processed with Mestrelab Research MNova software (version 14.2.1-27684). High-resolution mass spectrometry (HRMS) was performed on a 6540 Q-TOF LC/MS (Agilent Technologies, USA). The scanning electron microscopy (SEM) was performed on a JSM-7800F scanning electron microscope (JEOL Ltd., Japan). SERS (Surface-enhanced Raman scattering) spectra were recorded on a Renishaw inVia confocal Raman microscope (Renishaw, UK) using a 50× telephoto objective under 633 nm laser excitation and the data were processed using Renishaw WiRE (version 4.3) software.

1.3 Cell Culture.

Human T lymphocyte Jurkat clone E6-1 cells (Jurkat T) and Human breast cancer cell line MCF-7 were purchased from the Cell Bank of Type Culture Collection of Chinese Academy of Sciences (China). Jurkat T cells were cultured in RPMI-1640 media supplemented with 10% FBS, 2 mM GlutaMAX™ supplement, 1 mM sodium pyruvate (referred to as RPMI-1640 complete medium), at a density control of 1×10^{5}~1×10^{6} cells/mL. MCF-7 cells were cultured in RPMI-1640 media supplemented with 10% FBS, at a density control of 5×10^{5}~2×10^{6} cells/mL. Cells were cultured in a humidified incubator at 37 °C under 5% CO_2.

1.4 Sample preparation procedure for CLSM and STED imaging.

Confocal dishes were treated with 0.1% (w/v) poly-L-lysine solution (150 μL per well) for 1 h, followed by washing with 150 μL of PBS for 5 times, and drying. The prepared positively charged confocal dishes were stored in sterile environment. For imaging Jurkat T or MCF-7 cells, dispersed cells after staining were added to the treated dished and incubated for 15 min at r.t. prior to CLSM or STED imaging. Cells were observed under an oil-immersion 63x objective (CLSM imaging) or an oil-immersion 100x objective (STED imaging).

1.5 Sample preparation and analysis for FCM.

Cells were suspended in PBS buffer at 1×10^6 cells/mL and FCM was performed by counting 10,000 cells.

1.6 Synthesis.

Synthesis of chain transfer agent (CTA) 2-{[(butylsulfanyl)carbonothioyl]sulfanyl}propanoic acid (BTPA).

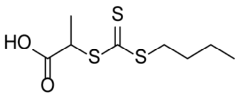

The synthesis procedure of this compound follows the previous report with slight modifications[1]. In a Schlenk flask, 1-butanethiol (purity 99%; 3.6 g, 40 mmol), 25% NaOH (purity 97%; 6.5 mL, 41 mmol) and carbon disulfide (purity ≥99.5%; 2.7 mL, 45 mmol) were mixed with 6.0 mL of water, followed by stirring for 30 min. To the obtained mixture, a solution containing 2-bromo-2-methylpropionic acid (purity 98%; 3.7 mL, 41 mmol) and 25% NaOH (6.0 mL, 38 mmol) was added slowly in an ice bath. After stirring overnight, 10 M HCl (purity 36.0-38.0%; 10 mL) was added to the reaction solution, followed by extraction with hexane. The organic phases were collected, dried over anhydrous magnesium sulfate, and concentrated in vacuo. After recrystallization from cyclohexane, a yellow crystalline solid was obtained (7.4 g, 77.7%).

^{1}H NMR (400 MHz, DMSO-d_6): δ (ppm) 13.16 (s, 1H), 4.68 (q, J = 7.3 Hz, 1H), 3.39 (t, J = 7.4 Hz, 2H), 1.63 (tt, J = 8.5, 6.8 Hz, 2H), 1.51 (d, J = 7.3 Hz, 3H), 1.43 – 1.31 (m, 2H), 0.89 (t, J = 7.3 Hz, 3H).

^{13}C NMR (100 MHz, DMSO-d_6): δ (ppm) 222.46, 171.52, 48.11, 36.19, 29.57, 21.40, 16.76, 13.41.

HRMS (ESI): m/z calculated for $C_8H_{15}O_2S_3$ $[M+H]^+$ 239.2435, found 239.2435.

Synthesis of DBCO-BTPA.

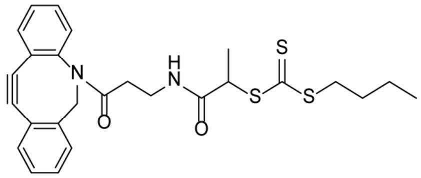

In a dry Schlenk flask, BTPA (35.7 mg, 0.15 mmol) and 1-hydroxybenzotriazole (purity ≥97%; 27.0 mg, 0.2 mmol) were combined in 2 mL of anhydrous DMF under nitrogen atmosphere and stirred for 10 min. Then DBCO-NH_2 (purity ≥94.5%; 27.6 mg, 0.1 mmol), fully dissolved in 1 mL of DMF in advance, was added dropwise to the above mixture. The solution was cooled to 0 °C in an ice-water bath, and then triethylamine (Et_3N, purity ≥99.5%; 28.9 μL, 0.2 mmol) and N-ethyl-N′-(3-dimethylaminopropyl) carbodiimide hydrochloride (EDC·HCl, purity ≥98.0%) (38.3 mg, 0.2 mmol) were added sequentially. After stirring overnight, the reaction mixture was diluted with ethyl acetate (EA, purity ≥99.7%), washed with water (3x), brine (3x), and dried over sodium sulfate (purity ≥99.0%). The volatiles were removed in vacuo and the crude product was purified by column chromatography (petroleum ether (PE, purity ≥99.7%)/EA = 1:1). The pure product was obtained as a yellow viscous oil (28.1 mg, 56.7%).

^{1}H NMR (400 MHz, $CDCl_3$): *δ* (ppm) 7.71 (d, *J* = 7.6 Hz, 1H), 7.45 – 7.22 (m, 7H), 6.53 (t, *J* = 6.2 Hz, 1H), 5.16 (d, *J* = 13.8 Hz, 1H), 4.50 (q, *J* = 7.3 Hz, 1H), 3.69 (d, *J* = 13.8 Hz, 1H), 3.41 – 3.22 (m, 4H), 2.47 (ddd, *J* = 16.6, 7.6, 4.3 Hz, 1H), 1.93 (ddd, *J* = 16.6, 7.0, 4.0 Hz, 1H), 1.73 – 1.63 (m, 2H), 1.48 (d, *J* = 7.2 Hz, 3H), 1.46 – 1.38 (m, 2H), 0.93 (t, *J* = 7.3 Hz, 3H).

^{13}C NMR (100 MHz, $CDCl_3$): *δ* (ppm) 223.03, 171.88, 170.18, 151.10, 148.09, 132.24, 129.21, 128.72, 128.51, 128.01, 127.36, 125.87, 123.12, 122.74, 115.05, 107.83, 55.67, 49.00, 37.16, 35.57, 34.77, 30.10, 29.85, 22.25, 16.85, 13.77.

HRMS (ESI): m/z calculated for $C_{26}H_{28}N_2O_2S_3Na$ $[M+Na]^+$ 518.9497, found 518.9497.

Synthesis of DBCO-SS-BTPA.

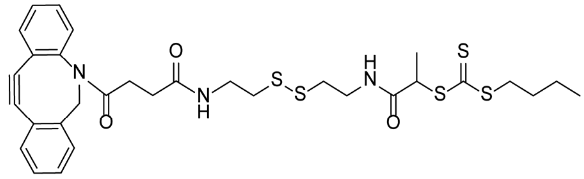

In a Schlenk flask, BTPA (35.7 mg, 0.15 mmol) and 1-hydroxybenzotriazole (27.0 mg, 0.2 mmol) were mixed in 1 mL of anhydrous DMF under nitrogen atmosphere and stirred well. To the obtained mixture, DBCO-SS-NH_2 (44.0 mg, 0.1 mmol) dissolved in DMF (1 mL) was added dropwise. The mixed solution was stirred in an ice-water bath for 15 min, and then Et_3N (28.9 μL, 0.2 mmol) and EDC·HCl (38.3 mg, 0.2 mmol) were sequentially added. After stirring for 12 h, the solution was diluted with EA, washed with water (3x), brine (3x) and dried over sodium sulfate. The crude product was spun dry in vacuo and purified using

column chromatography (PE/acetone (purity ≥99.7%) = 3:2) to finally obtain a faint yellow solid (29.7 mg, 45%).

^{1}H NMR (400 MHz, $CDCl_3$): δ (ppm) 7.67 (d, J = 7.3 Hz, 1H), 7.50 – 7.22 (m, 7H), 7.03 – 6.92 (m, 1H), 6.27 (s, 1H), 5.15 (d, J = 13.8 Hz, 1H), 4.71 (qd, J = 7.4, 4.3 Hz, 1H), 3.68 (d, J = 13.7 Hz, 1H), 3.54 (tq, J = 6.2, 3.2 Hz, 2H), 3.45 – 3.29 (m, 4H), 2.81 – 2.69 (m, 3H), 2.67 – 2.51 (m, 2H), 2.40 (ddd, J = 13.7, 8.7, 5.2 Hz, 1H), 2.26 (dddd, J = 14.9, 7.1, 5.4, 1.7 Hz, 1H), 2.05 – 1.95 (m, 1H), 1.72 – 1.63 (m, 2H), 1.54 (dd, J = 7.3, 2.6 Hz, 3H), 1.49 – 1.36 (m, 2H), 0.93 (td, J = 7.3, 1.3 Hz, 3H).

^{13}C NMR (100 MHz, $CDCl_3$): δ (ppm) 223.95, 172.64, 172.47, 170.93, 151.34, 148.15, 132.41, 129.39, 128.79, 128.42, 128.36, 127.95, 127.30, 125.69, 123.31, 122.66, 114.85, 108.01, 55.75, 48.28, 38.70, 38.50, 38.48, 37.73, 37.57, 37.36, 31.58, 30.82, 30.80,30.04, 29.85, 22.20, 16.49, 13.75.

HRMS (ESI): m/z calculated for $C_{31}H_{37}N_3O_3S_5Na$ $[M+Na]^+$ 681.9318, found 681.9318.

Synthesis of N-(2-hydroxypropyl) methacrylamide (HPMA).

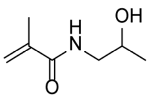

Under nitrogen atmosphere, amino-2-propanol (purity 99.5%; 2.34 mL, 0.03 mol), anhydrous triethylamine (4.2 mL, 0.03 mol) and dichloromethane (purity ≥99.7%, DCM) (12 mL) were added to a dry Schlenk flask in an ice bath. Under stirring, a premixed solution containing methacryloyl chloride (purity 98%; 3 mL, 0.03 mol) and DCM (6 mL) was added dropwise. After stirring overnight, the crude white solid product was concentrated in vacuo and further purified by column chromatography (EA) to obtain a white powder (4.1 g, 96%).

^{1}H NMR (400 MHz, D_2O): δ (ppm) 5.73 (s, 1H), 5.47 (s, 1H), 4.05 – 3.91 (m, 1H), 3.38 – 3.21 (m, 2H), 1.95 (s, 3H), 1.19 (d, J = 6.4 Hz, 3H).

^{13}C NMR (100 MHz, D_2O): δ (ppm) 172.15, 139.09, 121.01, 66.26, 46.24, 19.41, 17.67.

HRMS (ESI): m/z calculated for $C_7H_{14}NO_2$ $[M+H]^+$ 144.0493, found 144.0493.

Synthesis of methacrylamide-mannose (MA-Man) glycomonomer.

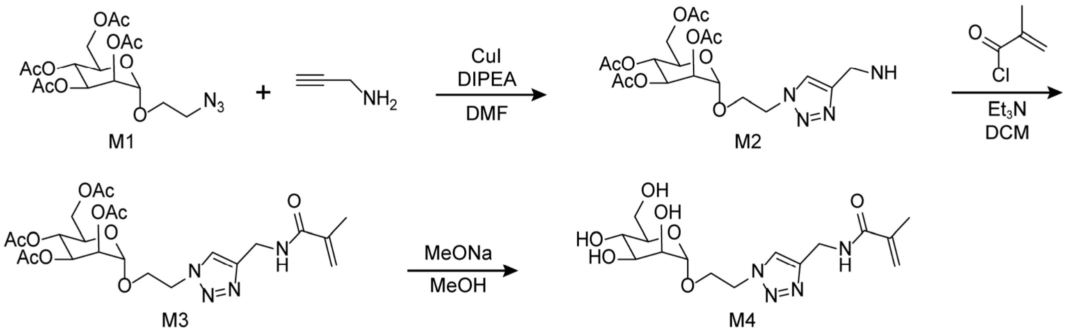

Azidomannose tetraacetate **M1** was synthesized following a previously reported protocol[2].

To a stirred solution of **M1** (0.834 g, 2 mmol) in DMF (5 mL), was added 0.26 mL of propargylamine (purity 98%; 4 mmol), 0.33 mL of N,N-diisopropylethylamine (DIPEA, purity 99%; 2 mmol) and 76 mg of CuI (purity ≥99.5%, 0.4 mmol). The reaction mixture was kept at r.t. for 12 h under nitrogen, and the mixture was concentrated in vacuo. The residue was dissolved in EA (100 mL) and washed with water (30 mL x 2) and brine (30 mL). The organic phase was dried over Na_2SO_4, filtered, evaporated to dryness in vacuo and used for next step without further purification.

To a stirred solution of **M2** (0.288 g, 0.6 mmol) in DCM (5 mL), was added 0.167 mL of Et_3N (1.2 mmol) and 64 μL of methacryloylchloride (0.66 mmol). The reaction mixture was kept at r.t. for 2 h under nitrogen, and the mixture was concentrated in vacuo. The residue was dissolved in EA (50 mL) and washed with water (10 mL x 2) and brine (10 mL). The organic phase was dried over Na_2SO_4, filtered, and evaporated to dryness in vacuo. The residue was purified by flash column chromatography on silica gel (PE/EA/Et_3N, 10:60:1) to afford **M3** (126 mg, 39%).

Compound **M3**:

^{1}H NMR (400 MHz, $CDCl_3$): *δ* (ppm) 7.61 (d, J = 4.0 Hz, 1H), 6.92 (d, J = 5.0 Hz, 1H), 5.70 (d, J = 4.7 Hz, 1H), 5.28 (t, J = 3.2 Hz, 1H), 5.18 – 5.12 (m, 1H), 5.09 – 5.05 (m, 1H), 4.69 (d, J = 5.6 Hz, 1H), 4.55 – 4.48 (m, 4H), 4.13 – 3.95 (m, 3H), 3.83 (dt, J = 10.3, 5.0 Hz, 1H), 3.50 – 3.42 (m, 1H), 2.06 (s, 3H), 2.02 (s, 1H) 1.97 (s, 3H), 1.92 (s, 3H), 1.90 (s, 3H).

^{13}C NMR (100 MHz, $CDCl_3$): *δ* (ppm) 170.53, 169.93, 169.60, 168.36, 144.91, 139.58, 123.38, 119.93, 97.35, 69.17, 69.01, 68.74, 66.17, 65.69, 62.19, 49.67, 35.15, 20.75, 20.68, 20.61, 20.60, 18.56.

HRMS (ESI): m/z calculated for $C_{23}H_{33}N_4O_{11}$ $[M+H]^+$ 541.2146, found 541.2147.

To a stirred solution of **M3** (230 mg, 0.4 mmol) in anhydrous CH_3OH (25 mL), was added $NaOCH_3$ (11 mg, 0.2 mmol) under an atmosphere of N_2 at r.t., and the stirring was continued at r.t. for 1 h. The reaction mixture was neutralized by adding acidic resins (Dowex 50W, hydrogen form), filtrated, concentrated in vacuo. The obtained **M4** was used in the following step without further purification (157 mg, 99%).

Compound **M4** (MA-Man):

^{1}H NMR (400 MHz, MeOD): *δ* (ppm) 7.88 (s, 1H), 5.75 – 5.73 (m, 1H), 5.41 – 5.38 (m, 1H), 4.71 (d, J = 1.6 Hz, 1H), 4.64 – 4.56 (m, 2H), 4.49 (s, 2H), 4.13 – 4.05 (m, 1H), 3.88 – 3.82 (m, 1H), 3.77 – 3.71 (m, 2H), 3.63 (dd, J = 11.8, 5.9 Hz, 1H), 3.57 (m, 2H), 3.16 – 3.10 (m, 1H), 1.95 (dd, J = 1.5, 1.0 Hz, 3H).

^{13}C NMR (100 MHz, MeOD): *δ* (ppm) 171.12, 146.31, 140.89, 125.02, 121.05, 101.47, 74.77, 72.40, 71.83, 68.33, 66.63, 62.68, 51.19, 35.85, 18.78.

HRMS (ESI): m/z calculated for $C_{15}H_{25}N_4O_7$ $[M+H]^+$ 373.1723, found 373.1720.

Synthesis of methacrylamide-galactose (MA-Gal) glycomonomer.

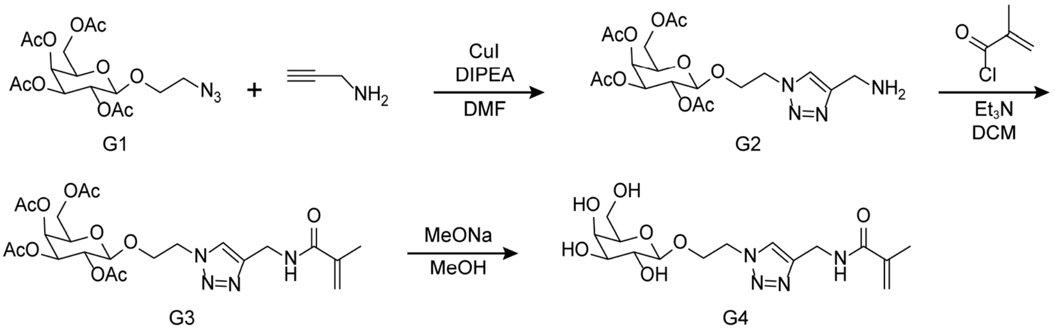

Azidogalactose tetraacetate **G1** was synthesized following a previously reported protocol[2]. To a stirred solution of **G1** (0.834 g, 2 mmol) in DMF (5 mL), was added 0.26 mL of propargylamine (4 mmol), 0.33 mL of DIPEA (2 mmol) and 76 mg of CuI (0.4 mmol). The reaction mixture was kept at r.t. for 12 h under nitrogen, and the mixture was concentrated in vacuo. The residue was dissolved in EA (100 mL) and washed with water (30 mL x 2) and brine (30 mL). The organic phase was dried over Na_2SO_4, filtered, evaporated to dryness in vacuo and used for next step without further purification.

To a stirred solution of **G2** (0.288 g, 0.6 mmol) in DCM (5 mL), was added 0.167 mL of Et_3N (1.2 mmol) and 64 μL of methacryloylchloride (0.66 mmol). The reaction mixture was kept at r.t. for 2 h under nitrogen, and the mixture was concentrated in vacuo. The residue was dissolved in EA (50 mL) and washed with water (10 mL x 2) and brine (10 mL). The organic phase was dried over Na_2SO_4, filtered, and evaporated to dryness in vacuo. The residue was purified by flash column chromatography on silica gel (PE/EA/Et_3N, 10:60:1) to afford **G3** (126 mg, 39%).

Compound **G3**:

^{1}H NMR (400 MHz, $CDCl_3$): δ (ppm) 7.60 (s, 1H), 6.72 (br, 1H), 5.77 (s, 1H), 5.39 (dd, J = 3.4, 0.9 Hz, 1H), 5.37 – 5.34 (m, 1H), 5.16 (dd, J = 10.5, 7.9 Hz, 1H), 4.98 (dd, J = 10.5, 3.4 Hz, 1H), 4.66 – 4.56 (m, 3H), 4.56 – 4.46 (m, 1H), 4.44 (d, J = 7.9 Hz, 1H), 4.25 (dt, J = 10.3, 3.9 Hz, 1H), 4.20 – 4.07 (m, 2H), 3.98 – 3.87 (m, 2H), 2.17 (s, 3H), 2.06 (s, 3H), 1.98 (s, 6H), 1.96 (s, 3H).

^{13}C NMR (100 MHz, $CDCl_3$): δ (ppm) 170.47, 170.23, 170.11, 169.65, 168.34, 144.40, 139.50, 123.74, 120.23, 100.94, 70.87, 70.56, 68.50, 67.67, 66.88, 61.23, 50.03, 35.17, 20.72, 20.70, 20.59, 18.64.

HRMS (ESI): m/z calculated for $C_{23}H_{33}N_4O_{11}$ $[M+H]^+$ 541.2146, found 541.2145.

To a stirred solution of **G3** (230 mg, 0.4 mmol) in anhydrous CH_3OH (25 mL), was added $NaOCH_3$ (11 mg, 0.2 mmol) under an atmosphere of N_2 at r.t., and the stirring was continued at r.t. for 1 h. The reaction mixture was neutralized by adding acidic resins (Dowex 50W, hydrogen form), filtrated, concentrated in vacuo. The obtained **G4** was used

in the following step without further purification (157 mg, 99%).

Compound **G4** (MA-Gal):

^{1}H NMR (400 MHz, MeOD): δ (ppm) 8.01 (s, 1H), 5.74 (s, 1H), 5.40 – 5.38 (m, 1H), 4.62 (t, J = 5.1 Hz, 2H), 4.49 (s, 2H), 4.27 (d, J = 7.2 Hz, 1H), 4.25 – 4.18 (m, 1H), 4.04 – 3.95 (m, 1H), 3.83 (d, J = 2.5 Hz, 1H), 3.79 – 3.67 (m, 2H), 3.57 – 3.44 (m, 3H), 1.94 (s, 3H).

^{13}C NMR (100 MHz, MeOD): δ (ppm) 170.92, 145.97, 140.89, 125.40, 120.99, 105.07, 76.74, 74.76, 72.29, 70.22, 68.98, 62.49, 51.57, 35.81, 18.77.

HRMS (ESI): m/z calculated for $C_{15}H_{25}N_4O_7$ $[M+H]^+$ 373.1723, found 373.1723.

1.7 CCK-8 assay of the cytotoxic effect of polymerization components.

Cell viability assay was performed according to the manufacturer's protocol. Briefly, Jurkat T cells of 1 × 10^6 cells/mL were respectively treated with HPMA monomer (r.t., 10 min), DBCO-BTPA (at 4 °C for 1 h), DBCO-SS-BTPA (at 4 °C for 1 h), and Fenton reagents (Fe^{2+}+H_2O_2; r.t., 10 min) of different concentrations.

Then the cells were separated by centrifugation and washed three times with phenol red-free RPMI-1640 to obtain cell pellets. The pellets, dispersed in complete medium, were seeded into 96-well plates (100 μL/well) at a density of 5 × 10^5 cells/mL (50,000 cells/well). The plates were cultured in a humidified incubator at 37 °C under 5% CO_2 for 24 h. After incubation, 10 μL of CCK-8 reagent was added to each well, and the cells were cultured for another 3.5 h. The absorption value (450 nm) of each well was analyzed using a Varioskan Flash spectral scanning multimode reader.

1.8 Solution Fenton-RAFT polymerization.

The polymerization medium was prepared by adding 100 μL B27 supplement minus AO to 4900 μL phenol-red free RPMI-1640, followed by adjusting the pH to 6.6~6.9. Native Jurkat T cells (2 × 10^6) were washed with phenol red-free RPMI-1640 and centrifuged to collect the cell pellet. Then, the pellet was dispersed in 100 μL of pre-deoxygenated polymerization medium. 120 μmol HPMA, 1.5 μmol BTPA and 0.45 μmol H_2O_2 were dissolved in 190 μL of polymerization medium, added to a glass vial and sealed, and the pre-reaction solution was bubbled with nitrogen for 30 min. The cell suspension was then quickly added to the deoxygenated pre-reaction solution, resealed, deoxygenated from the headspace for 3 min. To initiate the polymerization reaction in the solution, 10 μL of ammonium ferrous sulfate solution (purity 99%; 15 mM, prepared with pre-deoxygenated water) was injected into the reaction system. After polymerization for 1, 2, 5, or 10 min, the solution was immediately exposed to air and 700 μL of complete medium was added to terminate the polymerization. The reaction mixture was centrifuged at 4 °C. The cells were collected, washed 3 times with phenol red-free RPMI-1640 medium, and subjected to cell

viability and proliferation assays. The supernatant was added with D_2O for monomer conversion analysis by ^1H NMR.

1.9 Cell viability assay.

Cell viability assay using LIVE/DEAD™ Fixable Far-Red Dead Cell Stain Kit:

Prior to assay, cells were dispersed in 1 mL of PBS buffer at 1 x 10^6 cells/mL. Then 1 μL of LIVE/DEAD™ fixable far-red dead cell stains in DMSO were added to the cell suspension. The cells were incubated with the stains for 30 min at 4 °C. Then, the cell pellet was collected by centrifugation, washed, and resuspended in 1 mL of PBS. Cells were immediately analyzed using a CytoFLEX flow cytometer equipped with a 638 nm laser and 660/10 nm filters. 10,000 events were recorded. Cells killed by 75% ethanol were used as dead cell control.

Calcein-AM/PI double-staining experiment:

1×10^6 cells were stained in 1 mL of PBS containing 2 μM calcein AM and 4 μM PI at 37 °C for 15 min. After terminating the reaction by centrifugation and washing for 3 times, the cells were resuspended in PBS and analyzed using a CytoFLEX flow cytometer equipped with a 488 nm laser, 525/40 and 585/42 nm filters. The stained cell samples were also imaged using CLSM. For Calcein-AM, ex/em: 495/500-550 nm; for PI, ex/em: 540/580-630 nm.

1.10 Cell proliferation assay.

Cells suspended in RPMI-1640 complete medium were seeded in 96-well plates (100 μL/well) at a density of 3×10^5 (for cells collected from solution polymerization systems) or 5×10^5 (for cells after metabolic labeling, CTA installation, and in situ polymerization, respectively) cells/well. After culturing for 0, 24, 48 and 72 h in a humidified incubator at 37 ^oC under 5% CO_2, 10 μL of CCK-8 reagent was added to each well, and the cells were cultured for another 3.5 h. The absorption value (450 nm) of each well was analyzed using a Varioskan Flash spectral scanning multimode reader.

1.11 Measurement of the number of azides on the cell surface using ^{19}F NMR.

Jurkat T cells (2×10^5 cells/mL) were incubated with 40 μM of tetraacylated N-azidoacetylmannosamine ($Ac_4ManNAz$) in RPMI-1640 complete medium for 48 h in a humidified incubator at 37 ^oC under 5% CO_2 to obtain $Cell_{Sia}$-azide. Cells were then washed three times with PBS, dispersed at a concentration of 1 × 10^6 cells/mL in PBS containing 100 μM DBCO-PEG_3-F, and reacted at 4 °C for 5 h. Cells were washed three times with PBS and the number of cells was counted. After dispersing the cell pellet in 500 μL RIPA lysis buffer, the cells were completely lysed for 30 min, and the cell lysates were collected

for ^{19}F NMR. ^{19}F NMR samples were spiked with 10% v/v D_2O and CF_3COONa was used as the chemical shift reference.

1.12 SEM imaging of Cell-P.

The cell climbing pieces were immersed in 0.1% w/v poly-L-lysine solution, treated for 1 h at r.t., washed three times with PBS and dried at room temperature. The concentrated cell suspension was dropped onto the cell climbing pieces, and incubated at r.t. for 30 min. After aspirating non-adherent cells, the cell climbing pieces were washed once with PBS. The cells were fixed with 4% glutaraldehyde for 2 h at 4 °C and gently washed twice with PBS. The cells were dehydrated in succession with 30, 50, 70, 80, 90 and 100% ethanol for 5 min. The cell climbing pieces were then dried. Prior to SEM analysis, the samples were coated with gold for 100 s using an auto fine coater.

1.13 Intracellular reactive oxygen species assay.

1×10^6 cells were dispersed in 1 mL PBS containing 10 μM 2′,7′-dichlorodihydrofluorescein diacetate (DCFH-DA) and incubated at 37 °C for 30 min. After three washes with PBS, samples were analyzed by a CytoFLEX flow cytometer equipped with a 488 nm laser and a 525/40 nm filter, and imaged with CLSM (ex/em: 495/500-550 nm).

2. Supplementary Figures

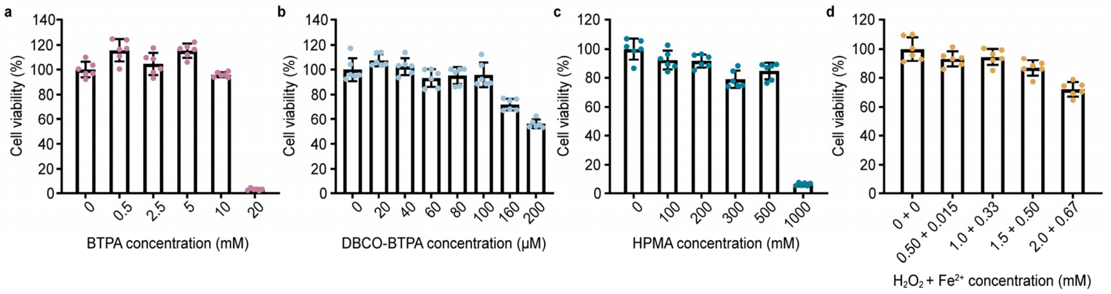

Supplementary Figure 1. Evaluation of the cytocompatibility of polymerization components. The viability of Jurkat T cells after treatment with (**a**) BTPA, (**b**) DBCO-BTPA, (**c**) HPMA, (**d**) Fenton reagents was measured using CCK-8 assay (*n*=6 independent samples, mean± SD). Data are representative of three independent experiments with similar results.

We confirmed that neither BTPA below 10 mM nor DBCO-BTPA below 100 μM had significant cytotoxicity (Supplementary Fig. 1a, b). 80% of Jurkat T cells remained active when HPMA was in the concentration range of 0 to 500 mM (Supplementary Fig. 1c). We selected the concentration of H_2O_2 as 1.5 mM and Fe^{2+} as 0.5 mM in the experiment, in which 87% of Jurkat T cells remained active (Supplementary Fig. 1d).

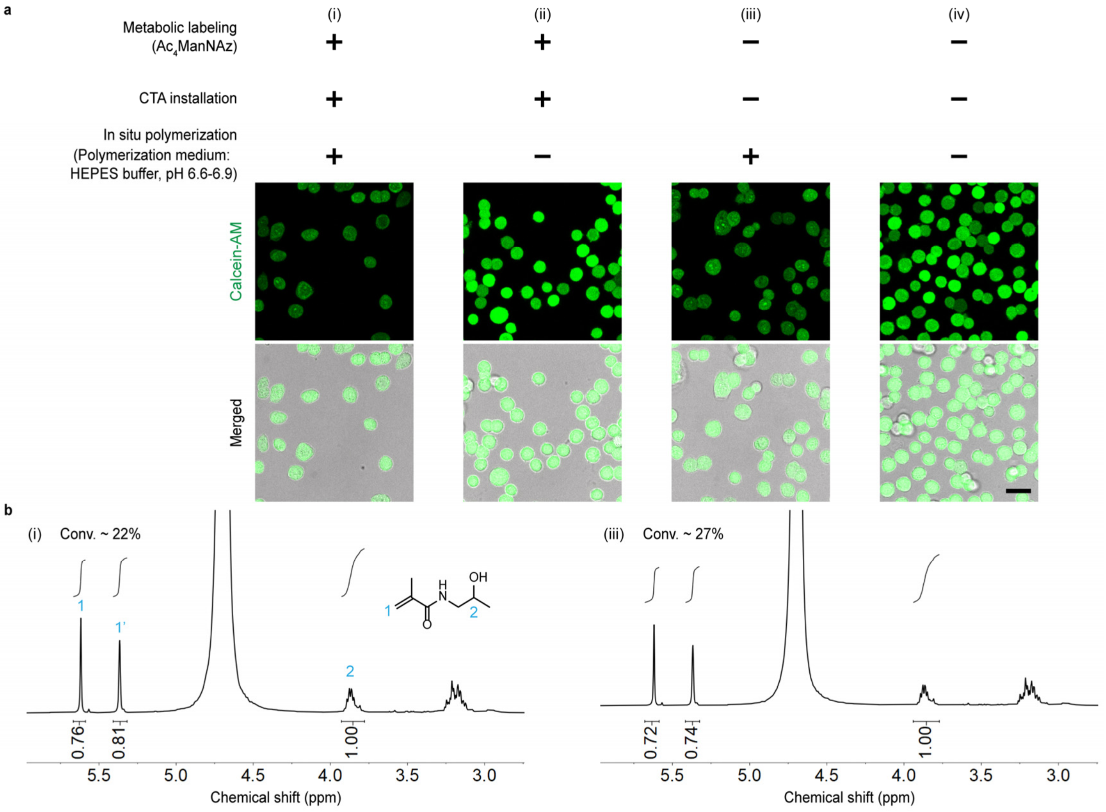

Supplementary Figure 2. Exclusion of the feasibility of using HEPES as a polymerization buffer for Jurkat T cells. The polymerization of each system (i~iv) was performed in HEPES buffer containing [HPMA]:[BTPA]:[H_2O_2]:[Fe^{2+}]=80:1:0.3:0.1 for 2 min. **a** Calcein-AM staining (green) to assess cell metabolic function. The fluorescence intensity is positively correlated with cell viability. Scale bar, 25 μm. **b** ^{1}H NMR spectra of the supernatants of the polymerization systems (i) cells with CTA installed, and (iii) cells without CTA installed. For each system, after polymerization, the solution was collected and centrifuged to obtain the supernatant. The monomer conversion (Conv.) was calculated by comparing the integrals of the unreacted vinyl protons (1 and 1') and the total monomer protons (2). Conv. = $(1 - I_{unreacted} / I_{total}) \times 100\%$, $I_{unreacted} = (I_1 + I_{1'}) / 2$. Data are representative of three independent experiments with similar results.

Although the polymerization reaction proceeded smoothly in HEPES buffer (>20% monomer conversion), the cell viability was found to be diminished compared to native cells by Calcein-AM staining.

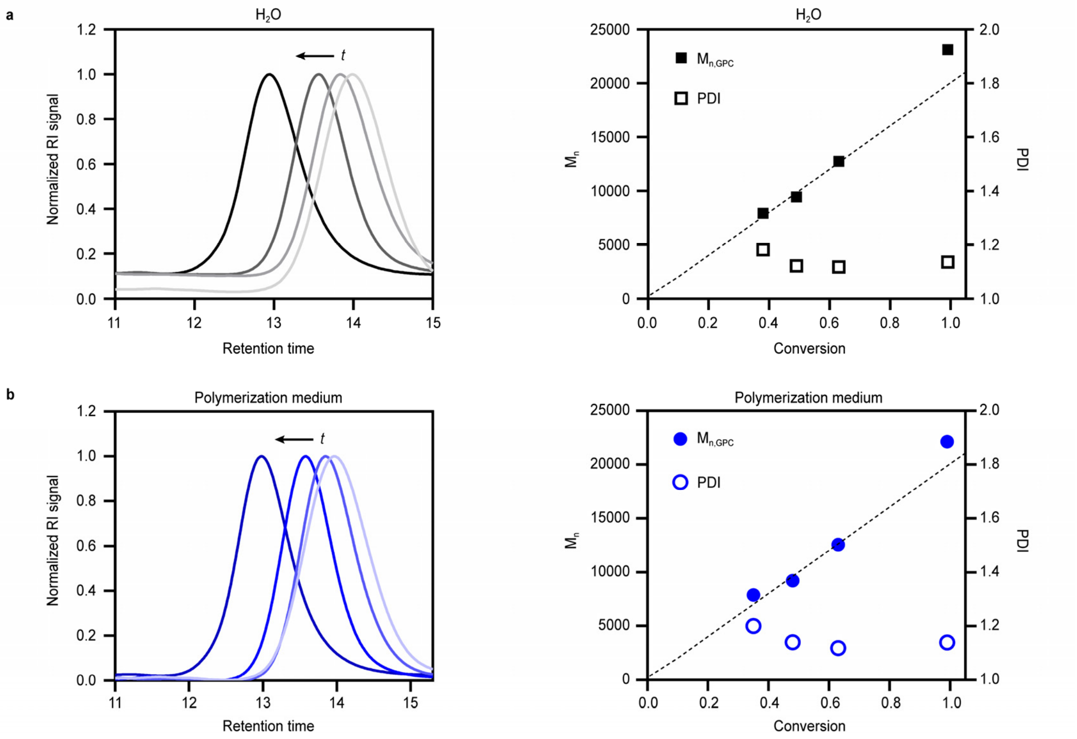

Supplementary Figure 3. Fenton-RAFT polymerization experiments in (**a**) water and (**b**) polymerization medium. GPC traces and plots of number average molecular weight (M_n) and polydispersity (PDI) versus monomer conversion are shown. The normalized refractive index (RI) signal was recorded with retention time. The arrows indicate longer polymerization time (*t*). The dotted line indicates the theoretical M_n calculated from the monomer conversions. The polymer was prepared under the condition of [DMA]:[BTPA]:[H_2O_2]:[Fe^{2+}] = 200:1:0.5:0.17 using the same polymerization apparatus and operations as the SSP protocol. Data are representative of three independent experiments with similar results.

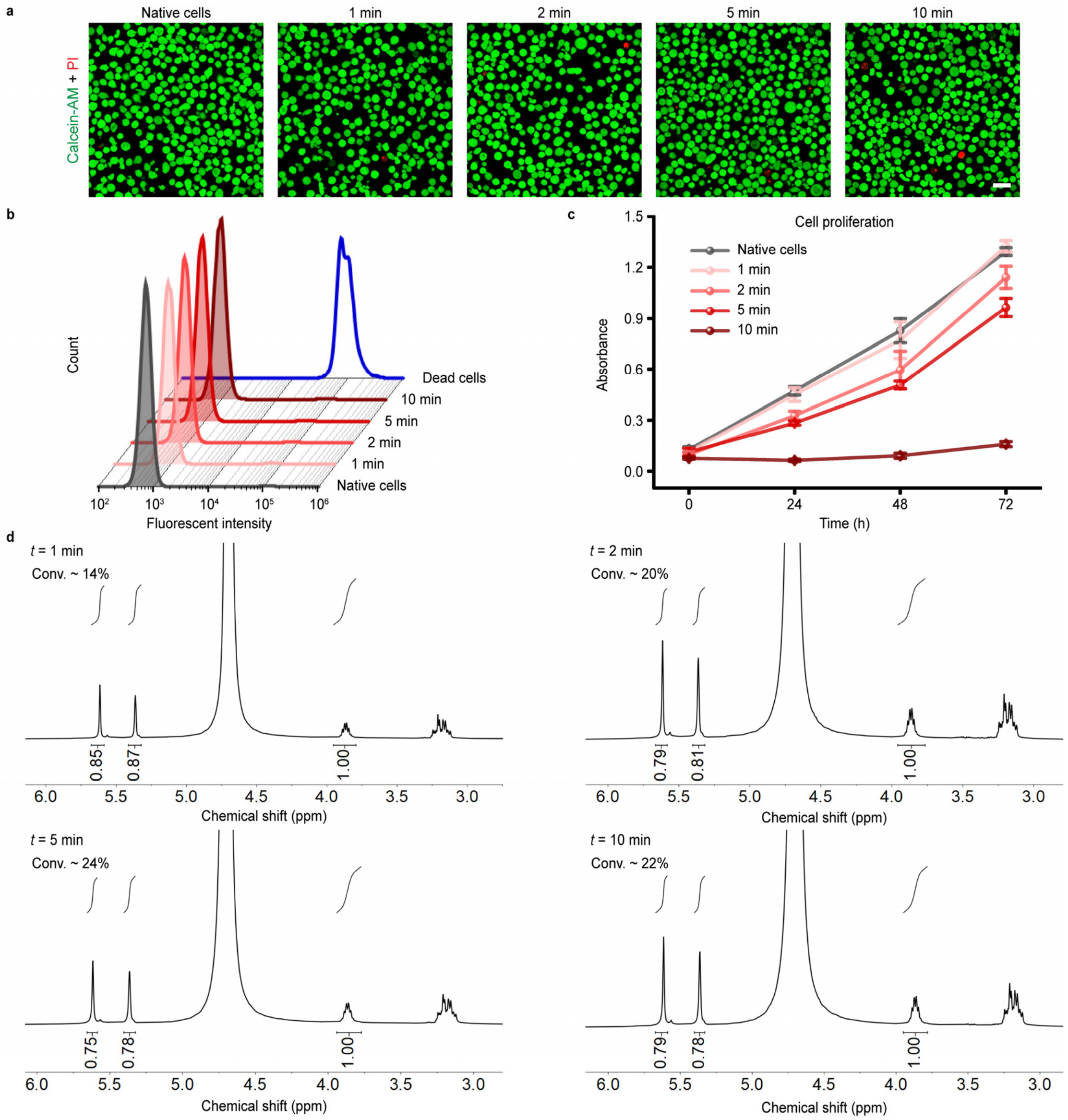

Supplementary Figure 4. Demonstration of the cytocompatibility and feasibility of solution polymerization. Polymerization buffer: phenol red-free RPMI-1640 containing B27 supplement minus AO. Jurkat T cells (without CTA installation) were suspended in the buffer, and the polymerization was performed under the condition of [HPMA]:[BTPA]:[H_2O_2]:[Fe^{2+}]=80:1:0.3:0.1 for 2 min. The effect of polymerization time (*t*, 1, 2, 5, 10 min) on cell viability, proliferation and monomer conversion was evaluated. **a** Calcein AM (viable cells, green) and propidium iodide (PI, dead cells, red) double-staining assay. Scale bar, 25 μm. **b** Cell membrane integrity assay by LIVE/DEAD^{TM} cell viability assay kit using flow cytometry. Live cells exhibit low fluorescence signal while dead cells show high fluorescence signal. Dead cell control was 75% ethanol-killed Jurkat T cells. The percentage of live cells was 98.7% (native cells), 98.4% (*t* = 1 min), 98.5% (*t* = 2 min), 98.3% (*t* = 5 min), and 98.3% (*t* = 10 min), respectively. **c** CCK-8 assay of cell proliferation ability after incubating cells, undergoing polymerization, for different periods of time (0, 24, 48, 72 h) (*n* = 6 independent samples, mean ± SD). **d** ^{1}H-NMR spectra of the supernatants of the systems with different polymerization time, for calculating monomer conversion (Conv.). Data are representative of three independent experiments with similar results.

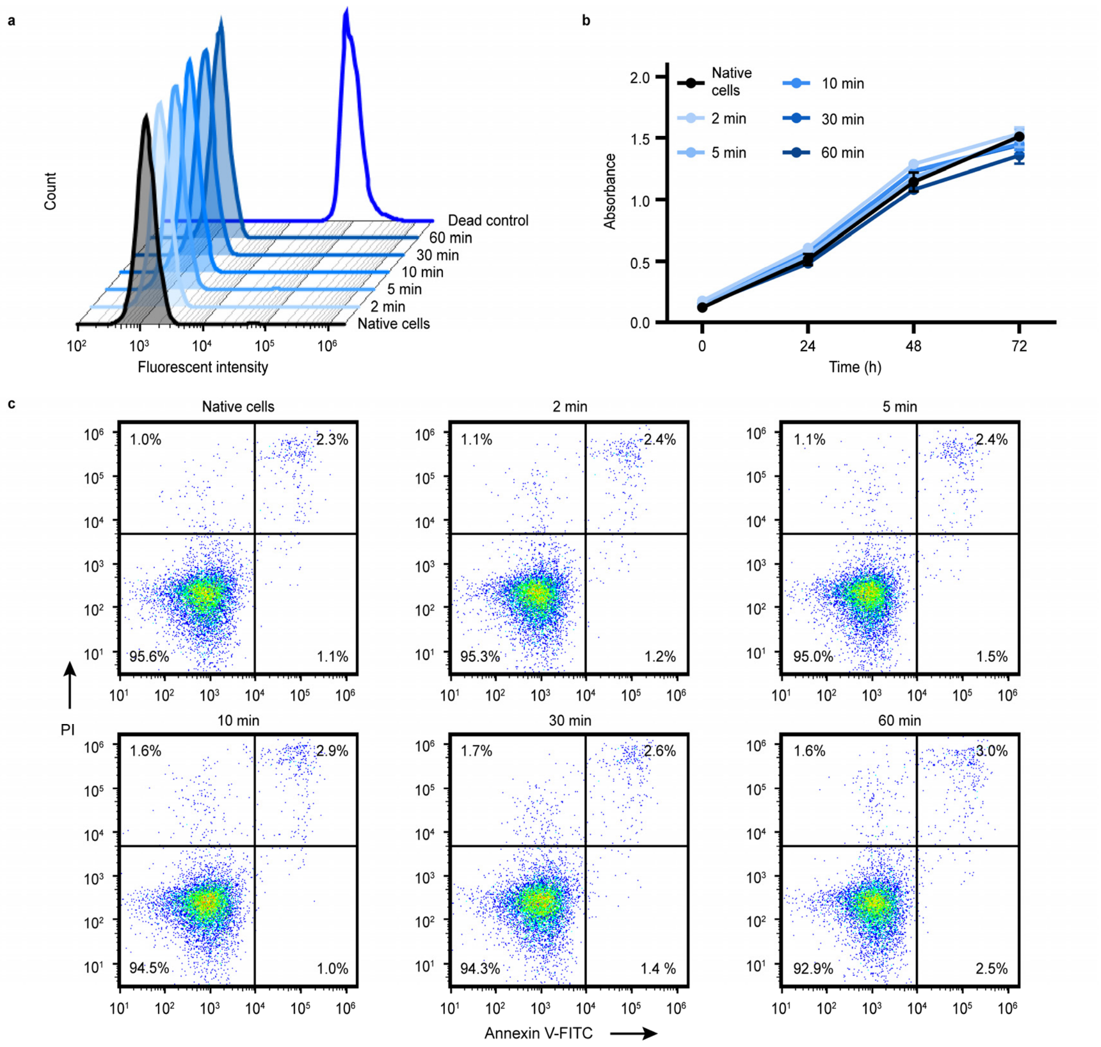

Supplementary Figure 5. Investigation of the effect of deoxygenation time on cell phenotype. Native Jurkat T cells were added to a polymerization medium that was pre-deoxygenated for 30 min and further deoxygenated for 2, 5, 10, 30 and 60 min. **a** Cell membrane integrity and cellular activity were measured by FCM using the LIVE/DEAD™ fixable far-red dead cell stain kit. Live control (native Jurkat T cells) and dead control (75% ethanol-killed Jurkat T cells) were also measured. The percentage of live cells was 99.2% (native cell), 99.5% (2 min), 99.2% (5 min), 99.1% (10 min), 99.3% (30 min), and 99.2% (60 min), respectively. **b** Cell proliferation was measured by CCK-8 kit after culturing treated or native cells for 0, 24, 48 and 72 h in the culture medium (n=6 independent samples, mean ± SD). **c** Apoptosis assay by FCM using Annexin V-FITC/propidium iodide (PI) apoptosis assay kit. There were no significant differences in cellular activity, proliferation capacity and apoptosis levels between native cells and cells after deoxygenation treatment for 2, 5, 10, 30 and 60 min, respectively. Data are representative of three independent experiments with similar results.

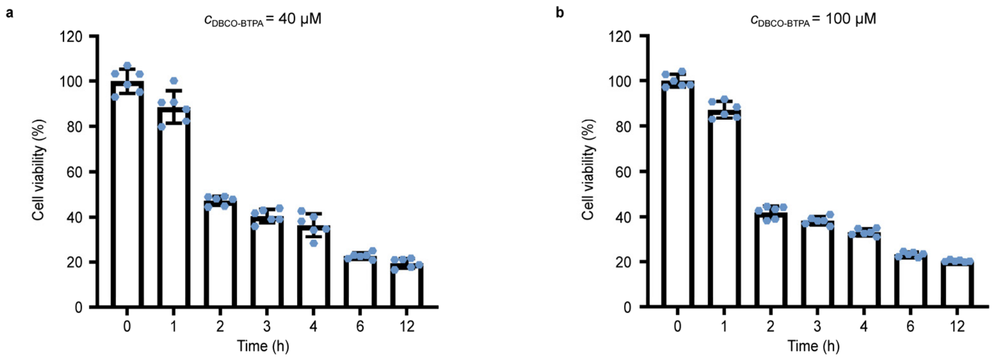

Supplementary Figure 6. Effect of the click reaction time of DBCO-BTPA on cell activity. The cell viability of Cell$_{\text{Sia}}$-azide after reaction with (**a**) 40 μM or (**b**)100 μM DBCO-BTPA for different time periods was measured by CCK-8 assay (*n*=6 independent samples, mean ± SD). $c_{\text{DBCO-BTPA}}$, concentration of DBCO-BTPA. Data are representative of three independent experiments with similar results.

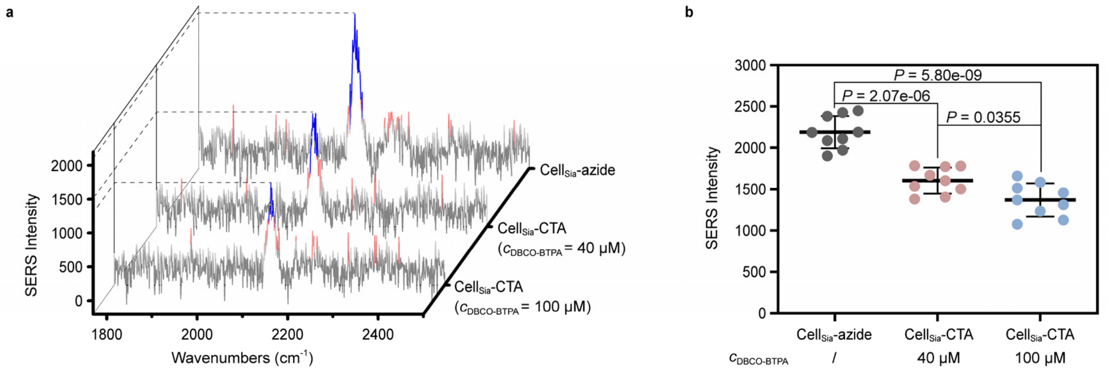

Supplementary Figure 7. SERS quantification of azide on cell surface after different treatments. The sialic acids on the surface of $Cell_{Sia}$-azide and $Cell_{Sia}$-CTA ($c_{DBCO\text{-}BTPA}$: 40 μM or 100 μM) were cleaved by neuraminidase. After centrifugation, the supernatants were collected and mixed with 15 nm gold nanoparticles (20 nM) in equal volume, followed by SERS detection of azide. **a** Representative SERS spectra. **b** Statistical data (n=9 independent measurements). Statistical differences were determined by one-way ANOVA with Tukey's multiple comparison test. $P < 0.05$ was considered statistically significant.

a

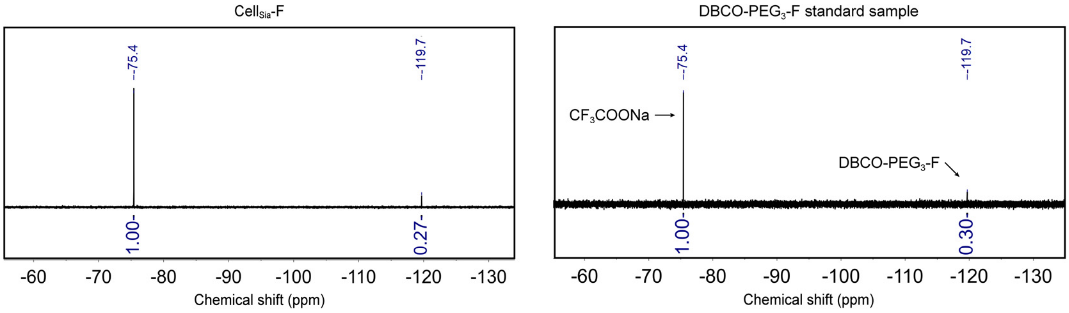

$$N_{azide} \text{ per Cell}_{Sia}\text{-azide} = \frac{5\times10^{-8}\times N_A\times0.27\times3}{N_{cells}} = 2.3\times10^{9}$$

b

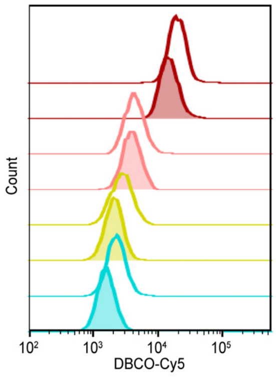

	MFI	N_{azide} per cell	N_{CTA} per cell
Cell$_{Sia}$-azide	18845	2.3×10^9	4.0×10^8
Cell$_{Sia}$-CTA	15350	1.9×10^9	
Cell$_{GalNAc}$-azide	3952	4.8×10^8	1.4×10^8
Cell$_{GalNAc}$-CTA	2763	3.4×10^8	
Cell$_{Met}$-azide	3324	4.0×10^8	1.4×10^8
Cell$_{Met}$-CTA	2158	2.6×10^8	
Cell$_{Cho}$-azide	2835	3.5×10^8	1.4×10^8
Cell$_{Cho}$-CTA	1755	2.1×10^8	

c

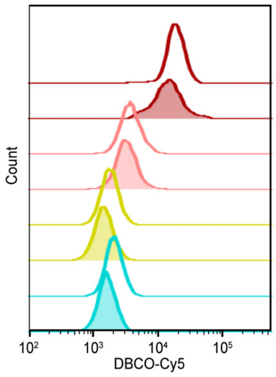

	MFI	N_{azide} per cell	N_{CTA} per cell
Cell$_{Sia}$-azide	17150	2.1×10^9	3.0×10^8
Cell$_{Sia}$-CTA	14869	1.8×10^9	
Cell$_{GalNAc}$-azide	2698	3.3×10^8	7.0×10^7
Cell$_{GalNAc}$-CTA	2153	2.6×10^8	
Cell$_{Met}$-azide	815	1.0×10^8	6.6×10^7
Cell$_{Met}$-CTA	280	3.4×10^7	
Cell$_{Cho}$-azide	948	1.2×10^8	6.7×10^7
Cell$_{Cho}$-CTA	433	5.3×10^7	

Supplementary Figure 8. Analysis of the number of azides or CTA on Cell-azide and Cell-CTA. **a** Jurkat cells were metabolically labeled with Ac_4ManNAz (generating Cell$_{Sia}$-azide) and then the azide tags were labeled with DBCO-PEG$_3$-F (generating Cell$_{Sia}$-F). The labeled cell samples were lysed and subjected to ^{19}F NMR measurement. The ^{19}F NMR spectrum of DBCO-PEG$_3$-F (100 μM) was also obtained with a chemical shift of -119.7 ppm. CF_3COONa (at -75.4 ppm) was used as the internal reference. In the equation, 5×10^{-8} is the amount of CF_3COONa in moles, N_A is Avogadro's constant, 0.27 is the integrated area of Cell$_{Sia}$-F (relative to CF_3COONa), and 3 is the ratio of F atom number in CF_3COONa to DBCO-PEG$_3$-F. N_{cells} is the total number of cells. **b** Flow cytometric measurements of Cell-azide and Cell-CTA. Cell samples were stained with DBCO-Cy5. The flow cytometric data can be used to calculate the azide number on different types of Cell-azide and Cell-CTA in combination with the azide number of Cell$_{Sia}$-azide. The difference between Cell-azide and Cell-CTA corresponds to the number of CTA. **c** Cell-azide and Cell-CTA were cultured for 24 h and stained with DBCO-Cy5, respectively, followed by flow cytometric analysis. The number of CTA on Cell-CTA after 24 h of culture was derived. MFI, mean fluorescence intensity. Data are representative of three independent experiments with similar results.

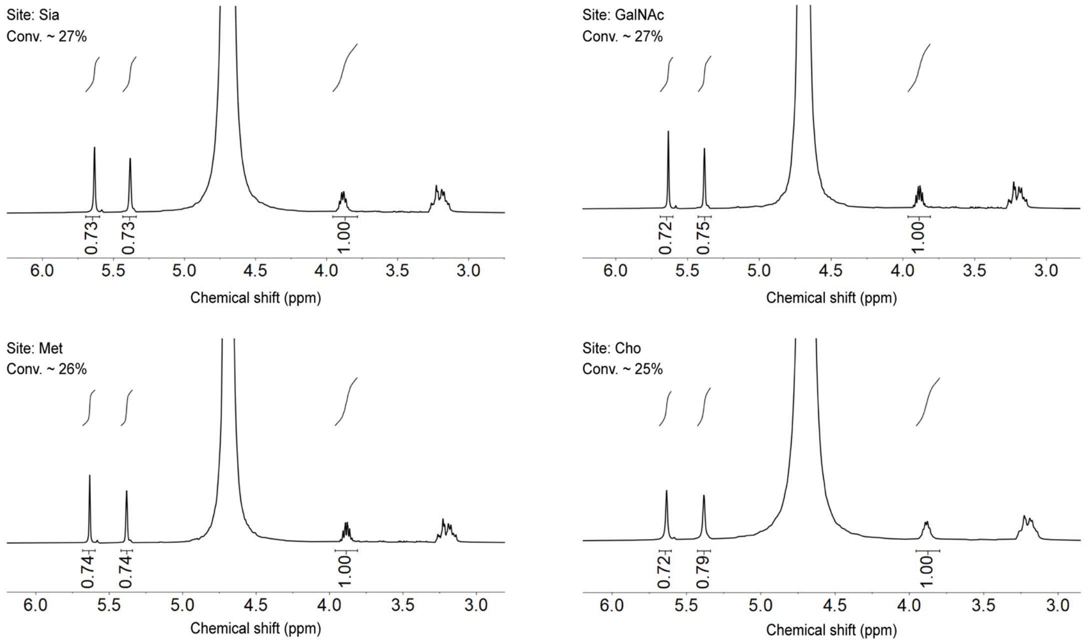

Supplementary Figure 9. Evaluation of the monomer conversion (Conv.) of different SSP systems with CTA respectively installed at Sia, GalNAc, Met or Cho sites of Jurkat T cells. After polymerization, the supernatant of each system was collected by centrifugation for ^{1}H NMR analysis. The Conv. of HPMA were calculated. Data are representative of three independent experiments with similar results.

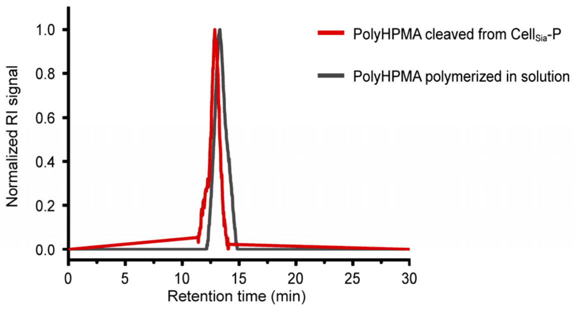

Supplementary Figure 10. GPC traces of polyHPMA cleaved from $Cell_{Sia}$-P, and polyHPMA synthesized in the same buffer (B-27 supplement minus AO was added to phenol red-free RPMI-1640, and the pH was adjusted to 6.6~6.9; no cell added). The normalized refractive index (RI) signal was recorded with retention time. Data are representative of three independent experiments with similar results.

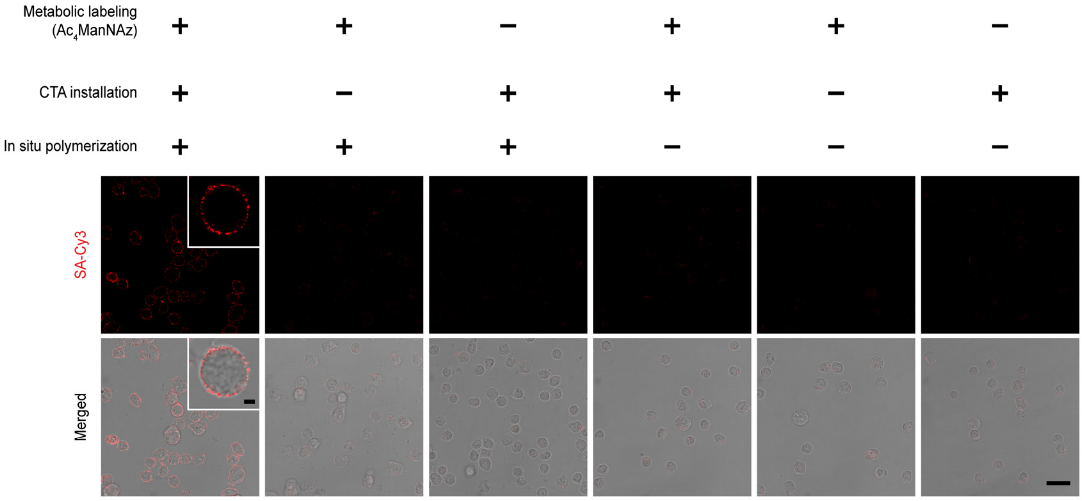

Supplementary Figure 11. Demonstration of the feasibility of in situ copolymerization using immobilized (dead) Jurkat T cells. Cells were subjected to in situ copolymerization of HPMA and AA-PEG_4-biotin (monomer ratio 7:1), and the biotin installed were stained by streptavidin-cyanine3 (SA-Cy3). Scale bar, 25 μm. Zoomed-in images showed a single stained cell. Scale bar, 3 μm. Data are representative of three independent experiments with similar results.

We examined different combinations of treatments and confirmed that the successful copolymerization is dependent on metabolic labeling, CTA anchoring, and initiation of polymerization, while ruling out the possibility that the fluorescence on the cell surface is derived from polymers in solution.

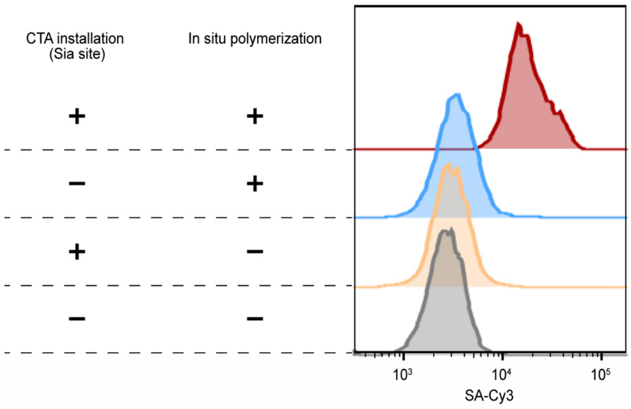

Supplementary Figure 12. In situ copolymerization of AA-PEG$_4$-biotin and HPMA at Sia sites generates P^{biotin} on the cell surface. The extent of SA-Cy3 binding to P^{biotin} on the cell surface was measured by FCM. Three control experiments were performed by omitting the CTA installation and/or polymerization step. Data are representative of three independent experiments with similar results.

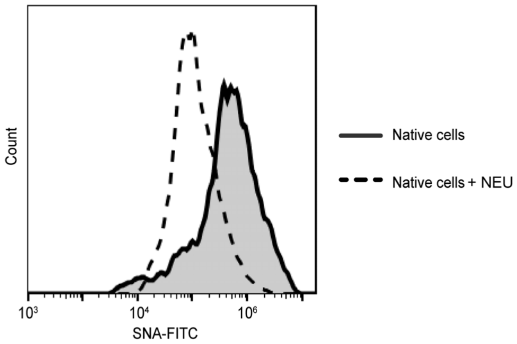

Supplementary Figure 13. Investigation of the Sia cleavage percentage by NEU on the surface of native cells. Jurkat cells with or without neuraminidase (NEU) treatment were incubated with SNA-FITC and then subjected to flow cytometry. The percentage of cleavage was calculated as ($MFI_{\text{native cells}}$ – $MFI_{\text{native cells + NEU}}$) / $MFI_{\text{native cells}}$, which was 68.6%. MFI, mean fluorescence intensity. Data are representative of three independent experiments with similar results.

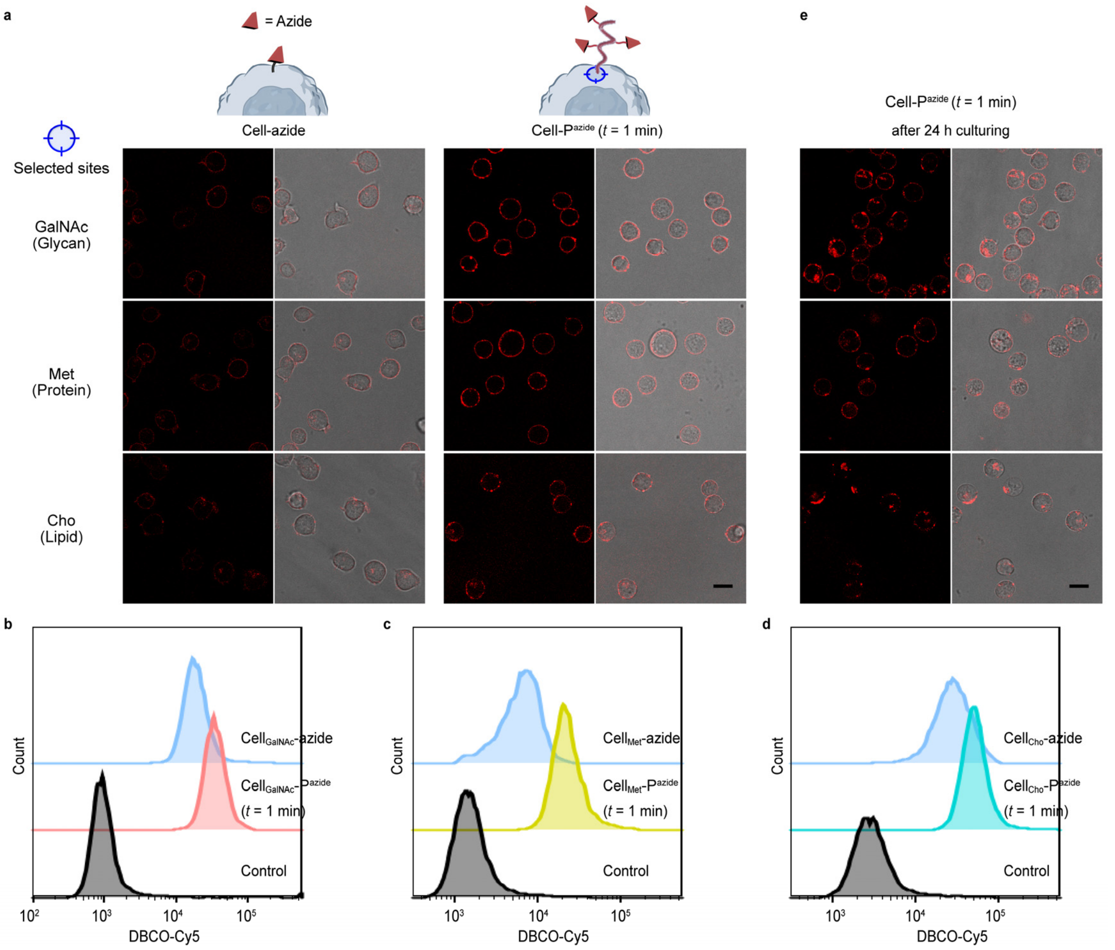

Supplementary Figure 14. In situ copolymerization (polymerization time *t*=1 min) of AA-PEG4-azide and HPMA at selected sites (GalNAc, Met or Cho). **a-d** Polymerized cells were stained with DBCO-Cy5 and then subjected to (**a**) CLSM imaging and (**b-d**) flow cytometric analysis. **e** To investigate the retention time of the polymers, DBCO-Cy5-stained Cell-P^{azide} were cultured for 24 h and then observed by CLSM. Scale bar, 10 μm. Data are representative of three independent experiments with similar results.

To evaluate the degree of polymerization at different polymerization times (*t* = 1 or 2 min), we calculated the MFI ratio of Cell-P^{azide} to Cell-azide in Fig. 3b-d and Supplementary Fig. 14b-d using the following equation:

Ratio = $(MFI_{Cell\text{-}P^{azide}} - MFI_{Control}) / (MFI_{Cell\text{-}azide} - MFI_{Control})$

The results were as follows:

GalNAc sites: 2.21 (*t*=2 min); 1.86 (*t*=1 min).

Met sites: 5.35 (*t*=2 min); 4.83 (*t*=1 min).

Cho sites: 3.96 (*t*=2 min); 2.02 (*t*=1 min).

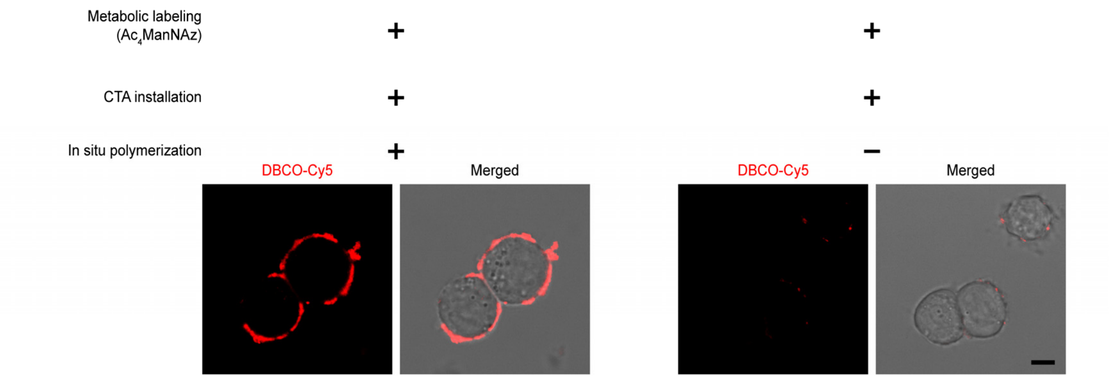

Supplementary Figure 15. Demonstration of the applicability of SSP strategy on MCF-7 cells. MCF-7 cells, a breast cancer cell line, were subjected to in situ copolymerization of HPMA and AA-PEG4-azide (monomer ratio 7:1), followed by DBCO-Cy5 staining. Cells only undergoing metabolic labeling and CTA installation were used as a control. Scale bar, 5 μm. Data are representative of three independent experiments with similar results.

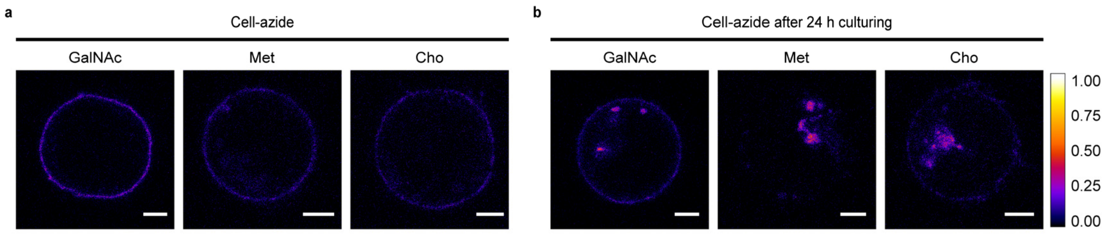

Supplementary Figure 16. Visualization of Cell-azide using STED. **a** STED images of single Cell-azide (sites: GalNAc, Met or Cho). Azide was stained with Click-iT™ sDIBO. **b** STED images of cells from (**a**) after 24 h of culture. Scale bar, 3 μm. Data are representative of three independent experiments with similar results.

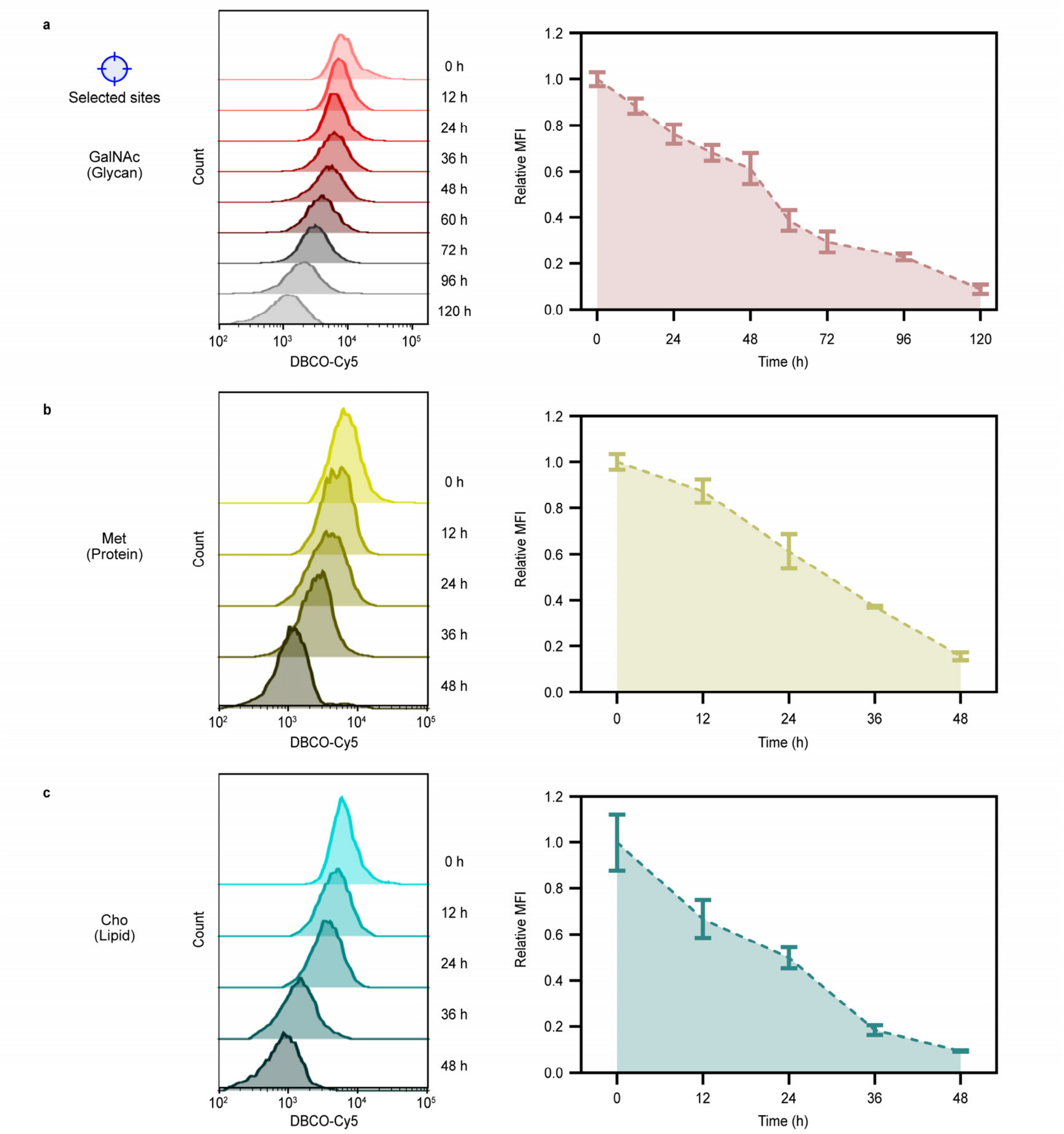

Supplementary Figure 17. Investigation of the retention time of the polymers. AA-PEG$_4$-azide and HPMA were in situ copolymerized (t = 2 min) at selected sites. **a** GalNAc. **b** Met. **c** Cho. After culturing in the complete medium at 37 °C for different periods of time, the Cell-P^{azide} were stained with DBCO-Cy5 for FCM analysis. Representative histograms were taken from three individual experiments. MFI, mean fluorescence intensity. For the three growth sites, MFI (relative to MFI at a culture time of 0 h) versus culture time was shown, and data were mean ± SD (n=3 individual experiments). Source data are provided as a Source Data file.

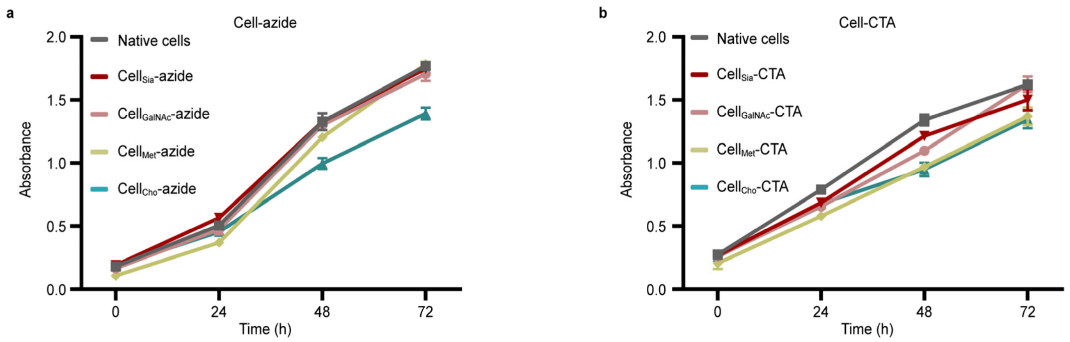

Supplementary Figure 18. Investigation of the cell proliferation ability. **a** Cell-azide. **b** Cell-CTA. Cell proliferation was measured using the CCK-8 kit after culturing native cells or each type of Cell-azide and Cell-CTA for 0, 24, 48, and 72 h in the culture medium (n = 6 independent samples, mean ± SD). Data are representative of three independent experiments with similar results. Source data are provided as a Source Data file.

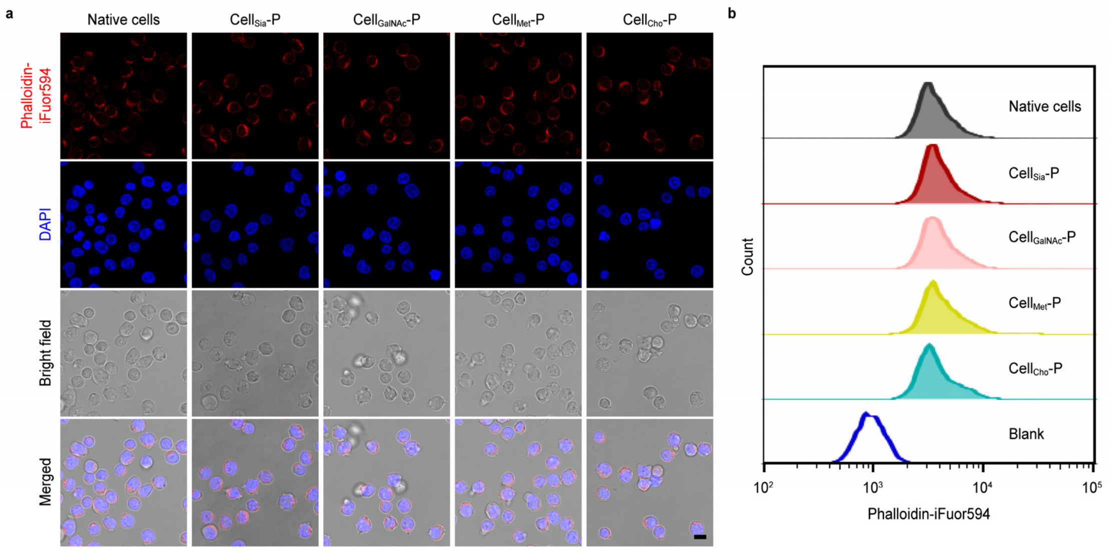

Supplementary Figure 19. Investigation of the effect of SSP on cellular actin cytoskeleton. **a** Staining of F-actin in different types of Cell-P and native Jurkat T cells with phalloidin-iFour594. Cell nuclei stained by 4′,6-diamidino-2-phenylindole dihydrochloride (DAPI). Scale bar, 10 μm. **b** Representative flow cytometry histograms. Data are representative of three independent experiments with similar results.

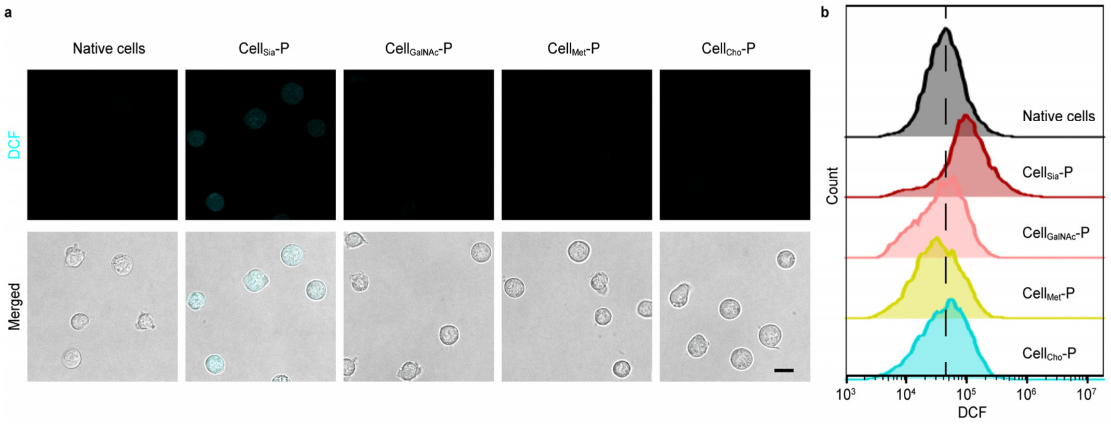

Supplementary Figure 20. Investigation of the effect of the SSP process on intracellular reactive oxygen species (ROS) levels. (**a**) CLSM and (**b**) FCM histograms of native cells and different types of Cell-P after 2′,7′-dichlorodihydrofluorescein diacetate (DCFH-DA) treatment were shown. Non-fluorescent DCFH-DA can be hydrolyzed by intracellular esterases to produce DCFH, which can be oxidized by intracellular ROS to produce fluorescent 2′,7′-dichlorofluorescein (DCF). Scale bar, 10 μm. Data are representative of three independent experiments with similar results.

The intracellular ROS levels of $Cell_{GalNAc}$-P, $Cell_{Met}$-P, or $Cell_{Cho}$-P were comparable to those of native cells. The slight increase in intracellular ROS concentration in $Cell_{Sia}$-P was probably due to the fact that the amount of the CTA anchored to Sia sites was three times that of the other sites (see Supplementary Fig. 8).

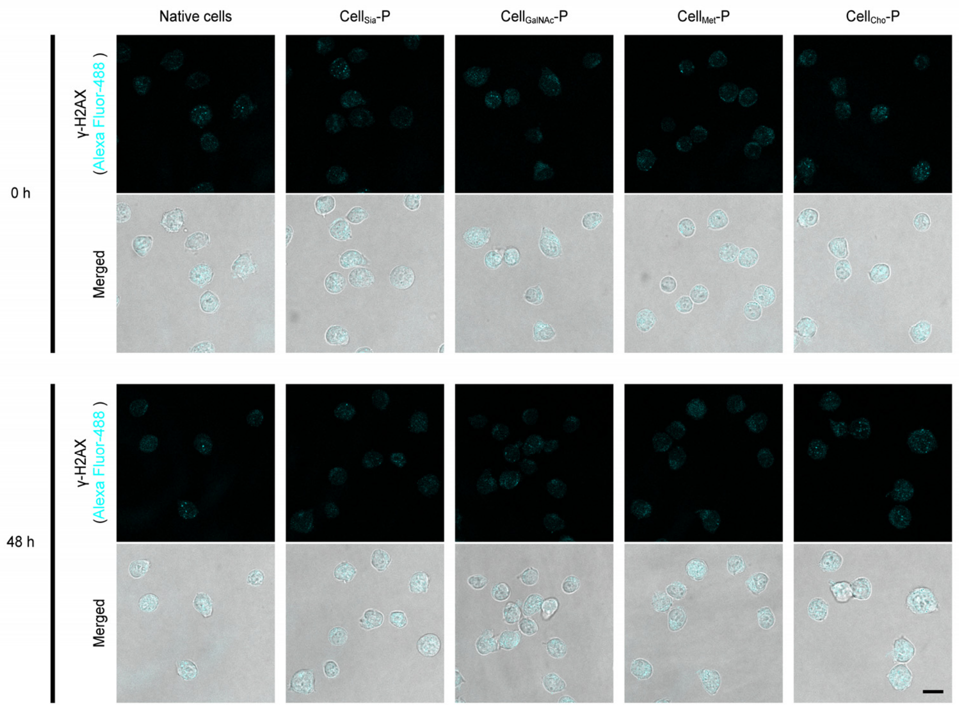

Supplementary Figure 21. Investigation of the effect of the SSP process on the level of intracellular DNA damage. CLSM imaging of the immunostaining signals of γ-H2AX in Cell-P immediately after polymerization and after 48 h of culture, respectively. Native cells were used as controls. Scale bar, 10 μm. The expression level of the phosphorylated histone H2AX (γ-H2AX) represents the extent of DNA damage. Data are representative of three independent experiments with similar results.

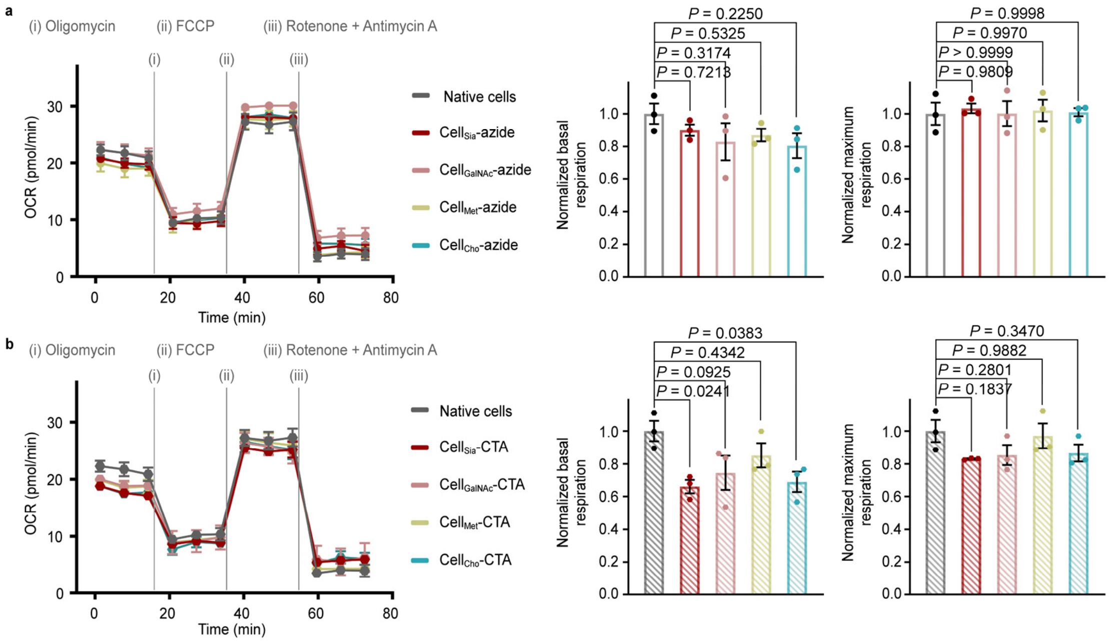

Supplementary Figure 22. Investigation of the effect of metabolic labeling and CTA anchoring on cellular metabolism. Oxygen consumption rates (OCRs) of cells were measured using the Seahorse XF Cell Mito Stress Test, and statistically normalized to basal and maximal respiration levels (n = 3 independent samples, mean ± SEM). **a** Cell-azide. **b** Cell-CTA. The same set of native cells was used as a control in (**a**), (**b**) and Fig. 4g. Statistical differences were determined by one-way ANOVA with Tukey's multiple comparison test. $P < 0.05$ was considered statistically significant. Data are representative of three independent experiments with similar results. Source data are provided as a Source Data file.

Oxygen consumption was assessed by measuring basal OCRs followed by the sequential addition of (i) oligomycin (an inhibitor of ATP synthesis), (ii) carbonyl cyanide-4 (trifluoromethoxy) phenylhydrazone (FCCP, uncoupling ionophore), and (iii) rotenone + antimycin A (blocking agents for complexes I and III of the electron transport chain, respectively) to evaluate the contribution of mitochondrial and non-mitochondrial mechanisms to oxygen depletion.

The OCR profiles of Cell-azide and Cell-CTA were similar to those of native cells. The basal respiration capacity of Cell-azide was unaffected by metabolic labeling. After CTA installation, the basal respiration levels of $Cell_{Sia}$-CTA and $Cell_{Cho}$-CTA were slightly reduced, and $Cell_{GalNAc}$-CTA and $Cell_{Met}$-CTA were not significantly different from native cells.

After decoupling of the mitochondrial membrane using FCCP, a robust increase in OCR was observed for both Cell-azide and Cell-CTA, with no significant difference in maximal respiration capacity compared to native cells.

Taken together, these data indicate an excellent basal and maximal respiration capacity of the cells after metabolic labeling and CTA anchoring.

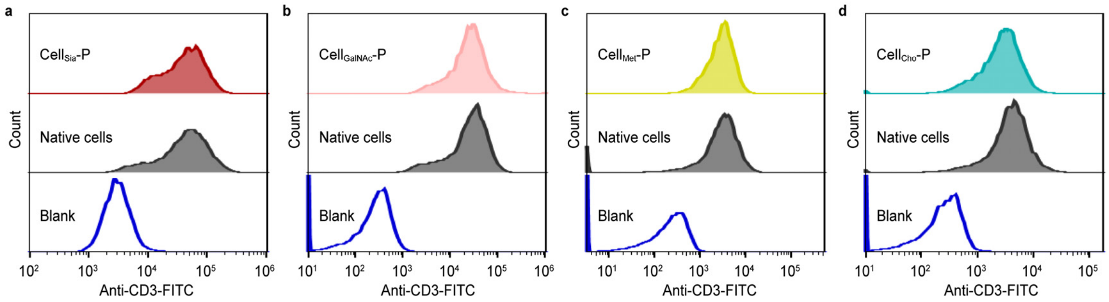

Supplementary Figure 23. Investigation of the effect of SSP on the accessibility of CD3 on the surface of Jurkat T cells. FCM histograms for immunostaining of CD3 on cell surface using anti-CD3-FITC are shown. **a** Cell$_{Sia}$-P. **b** Cell$_{GalNAc}$-P. **c** Cell$_{Met}$-P. **d** Cell$_{Cho}$-P. Data are representative of three independent experiments with similar results.

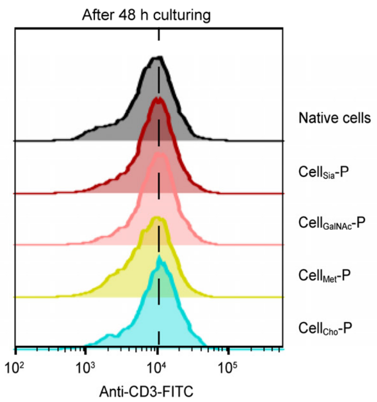

Supplementary Figure 24. Investigation of the antibody accessibility to Cell-P after 48 h culture. Representative FCM histograms of different types of Cell-P after 48 h culture and incubation with anti-CD3-FITC. Native cells were used as controls. The binding capacity of different types of Cell-P after 48 h culturing to anti-CD3 antibody was comparable to that of native cells, regardless of the CTA installation site. Data are representative of three independent experiments with similar results.

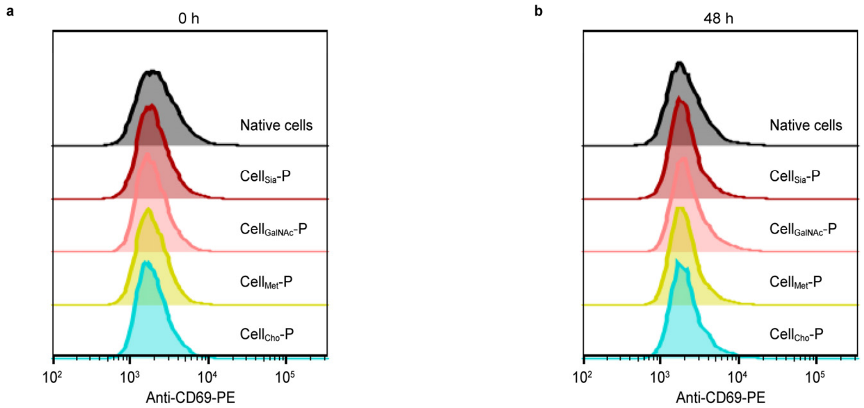

Supplementary Figure 25. Investigation of the effect of the SSP process on T cell activation. Representative FCM histograms of different types of Cell-P (**a**) immediately after polymerization and (**b**) after 48 h of culture, respectively, followed by incubation with anti-CD69-PE. Native cells were used as controls. Data are representative of three independent experiments with similar results.

In both cases, there was no significant difference between different types of Cell-P and native cells, suggesting that the SSP process does not lead to aberrant activation of Cell-P.

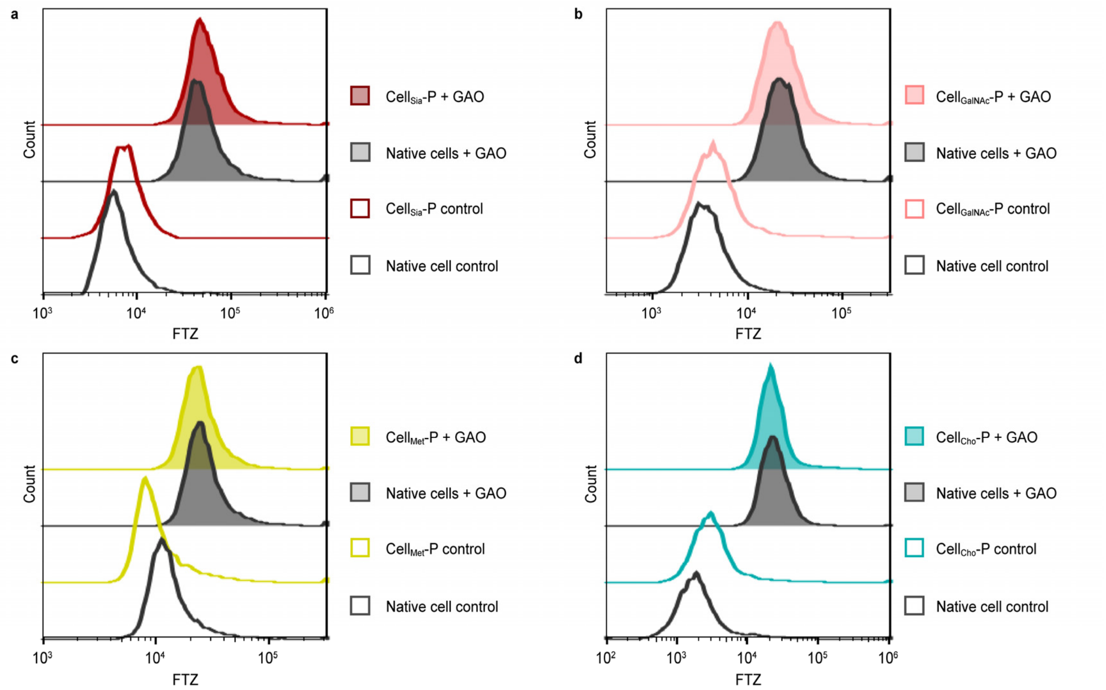

Supplementary Figure 26. Investigation of the effect of SSP on the GAO oxidation of cell surface Gal/GalNAc. Cells with polymer grown at different sites were treated with and without GAO (control), followed by fluorescein-5-thiosemicarbazide (FTZ) staining of the generated aldehyde groups and FCM analysis. **a** $Cell_{Sia}$-P. **b** $Cell_{GalNAc}$-P. **c** $Cell_{Met}$-P. **d** $Cell_{Cho}$-P. Native cells were used for comparison in each group. Data are representative of three independent experiments with similar results.

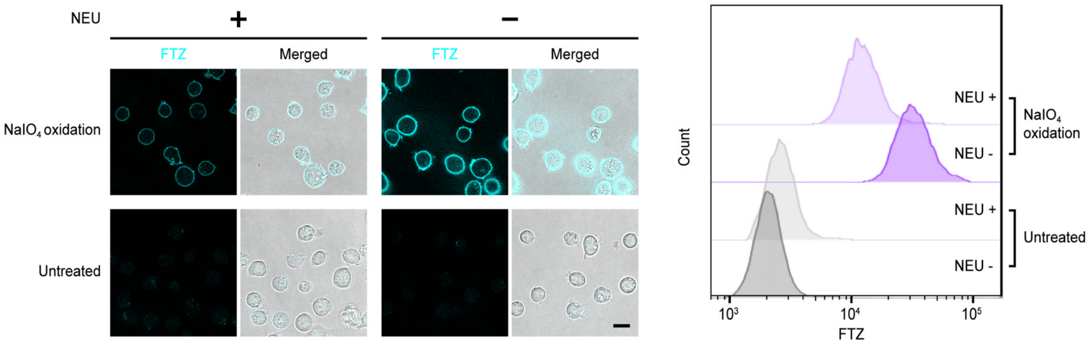

Supplementary Figure 27. Demonstration of the specificity of $NaIO_4$ oxidation of cell surface Sia. CLSM images and FCM histograms of native cells treated with or without $NaIO_4$, cleaved by neuraminidase (NEU) or not, and stained with fluorescein-5-thiosemicarbazide (FTZ). Scale bar, 10 μm. Data are representative of three independent experiments with similar results.

The percentage of cleavage was 65.9%, comparable to that of native cells (Supplementary Fig. 13), thus demonstrating that cell surface Sia can be specifically oxidized under optimal experimental conditions (1 mM $NaIO_4$, 4 °C, 15 min).

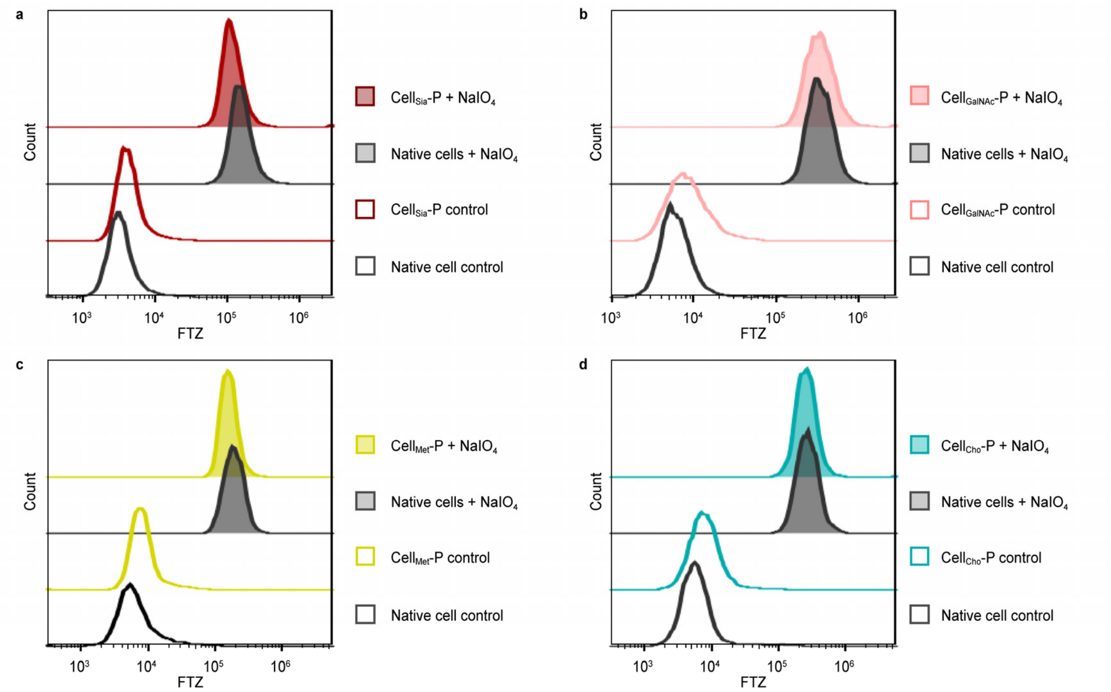

Supplementary Figure 28. Investigation of the effect of SSP on the oxidation of terminal Sia on Jurkat T cell surface by small molecule $NaIO_4$. Cells with polymer grown at different sites were treated with and without $NaIO_4$ (control), followed by FTZ staining and FCM analysis. **a** $Cell_{Sia}$-P. **b** $Cell_{GalNAc}$-P. **c** $Cell_{Met}$-P. **d** $Cell_{Cho}$-P. Native cells were used for comparison in each group. Data are representative of three independent experiments with similar results.

After $NaIO_4$ treatment, only the $Cell_{Sia}$-P group displayed obvious decrease compared with the native cells.

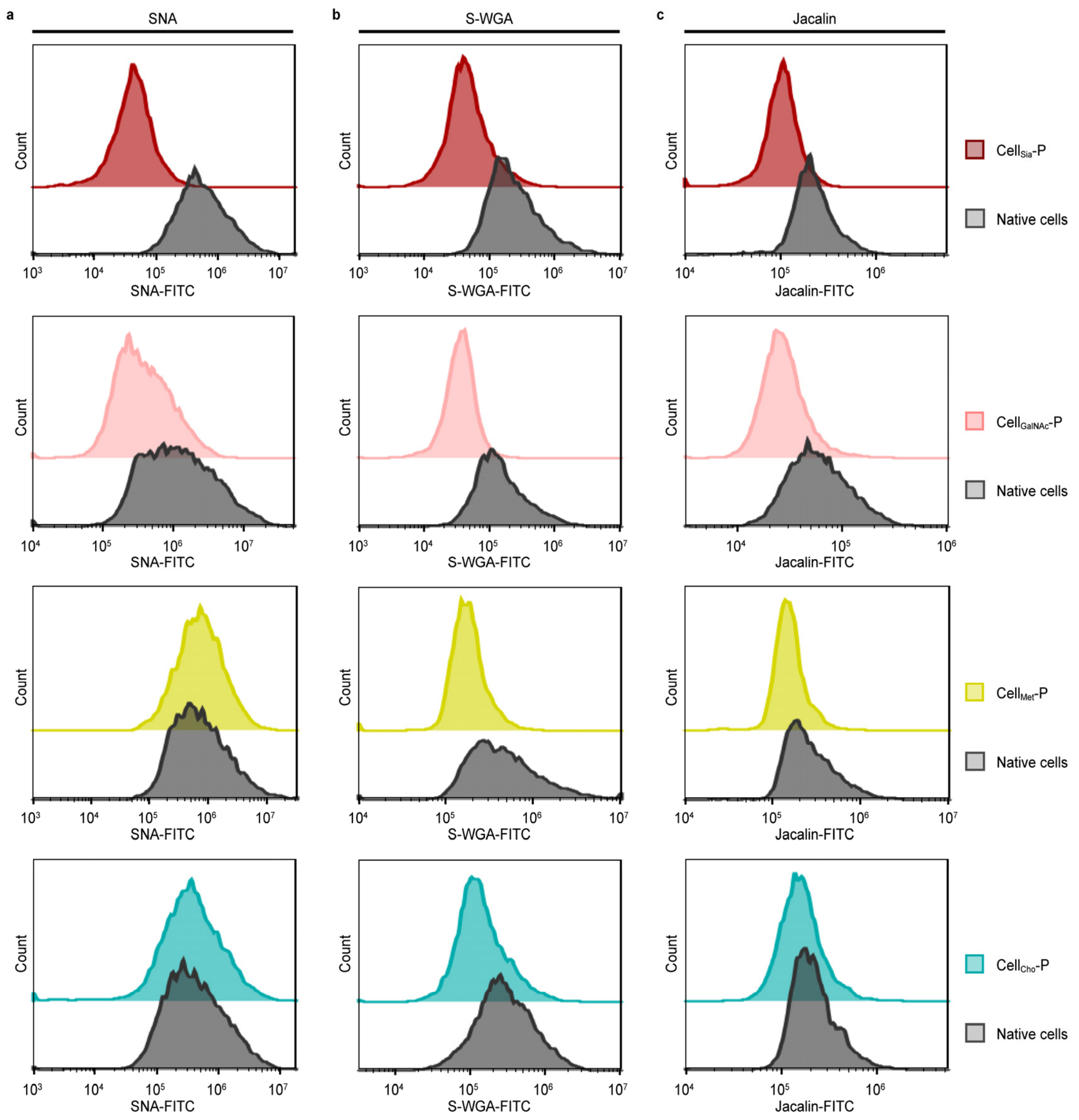

Supplementary Figure 29. The effect of SSP on the multivalent recognition between cell surface glycans and exogenous lectins depends on the polymerization site. Representative FCM histograms of Cell-P and native cells after incubation with different lectins. **a** Sambucus Nigra lectin (SNA). **b** succinylated wheat germ agglutinin (S-WGA). **c** Jacalin. Data are representative of three independent experiments with similar results.

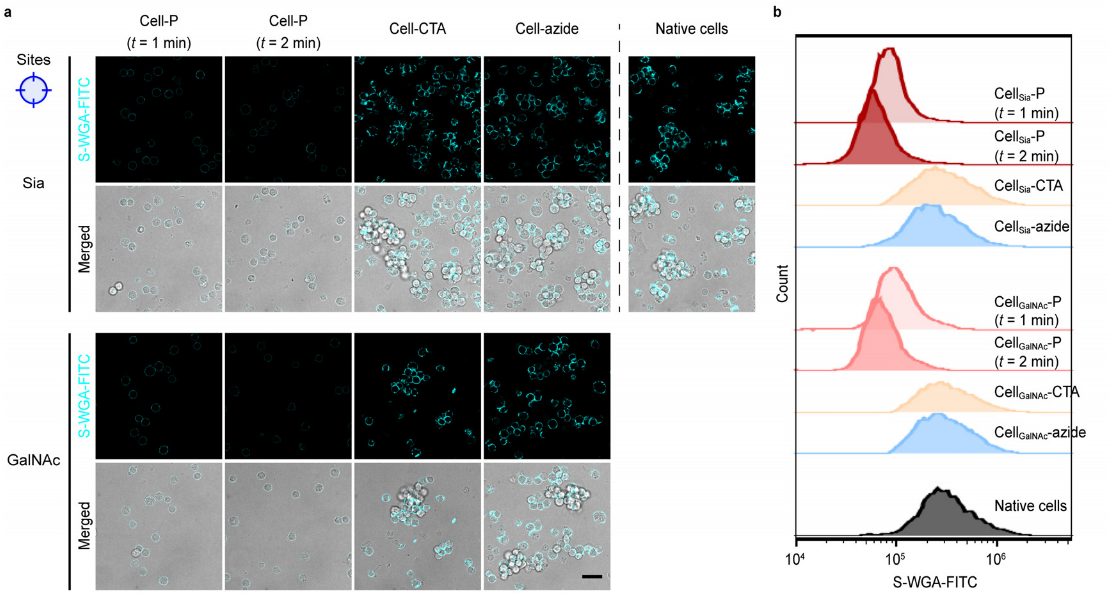

Supplementary Figure 30. Investigation of the extent of succinylated wheat germ agglutinin (S-WGA) binding to Cell$_{Sia}$-P and Cell$_{GalNAc}$-P obtained by polymerization for 1 or 2 min. (**a**) CLSM images and (**b**) FCM histograms of Cell$_{Sia}$-P and Cell$_{GalNAc}$-P after incubation with S-WGA-FITC were shown. Native cells, as well as Cell-azide and Cell-CTA (Sia or GalNAc sites) were used as controls. Scale bar, 25 μm. Data are representative of three independent experiments with similar results.

Blocking of lectin binding was observed in all the Cell$_{Sia}$-P and Cell$_{GalNAc}$-P samples. Longer polymerization time may lead to higher polymer molecular weight, resulting in greater hindering effect against the lectin.

In addition, Cell-azide and Cell-CTA bound lectin to a similar extent as native cells, indicating that metabolic labeling and CTA anchoring did not affect lectin binding.

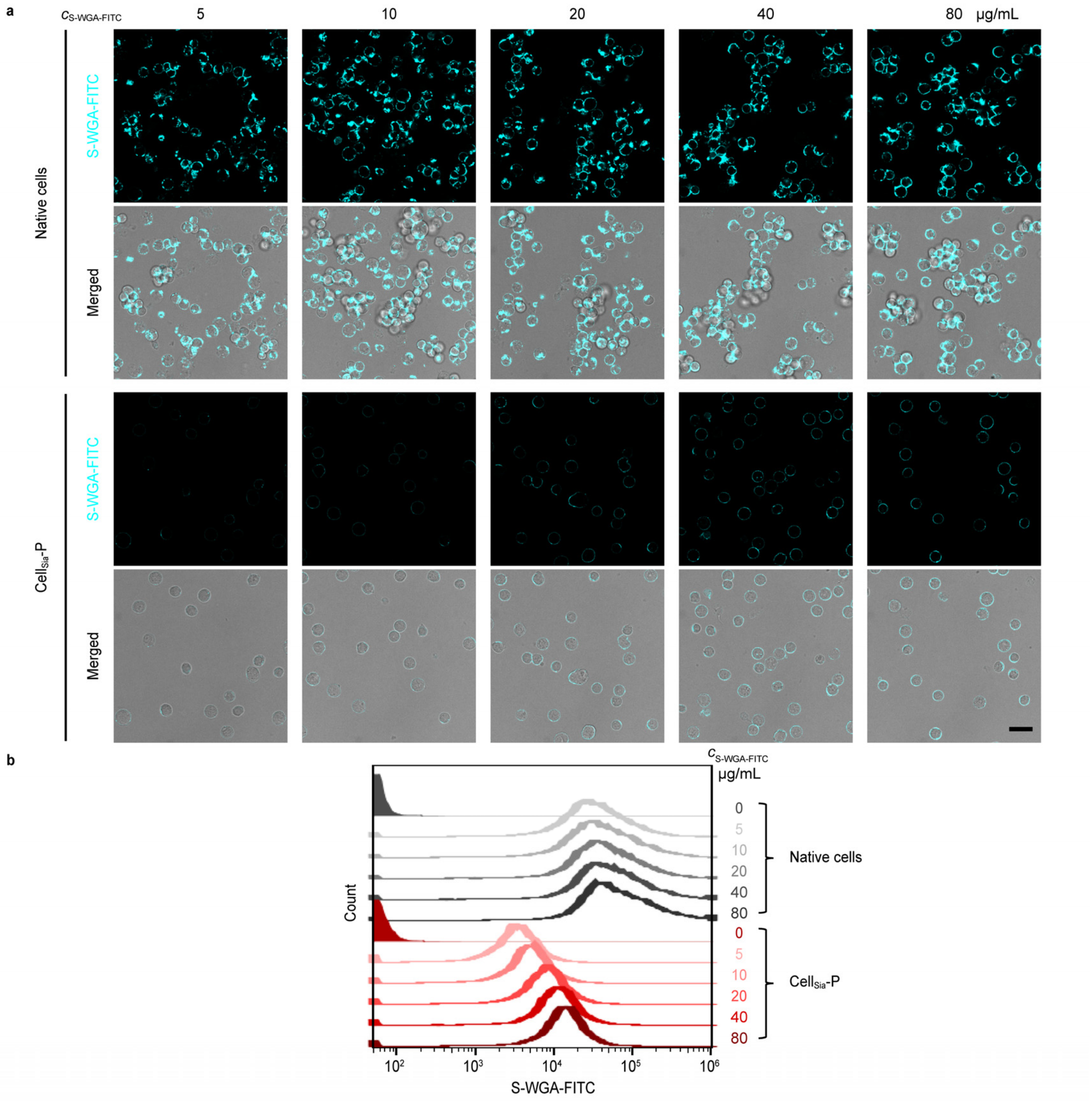

Supplementary Figure 31. Saturation binding assay. Cell$_{Sia}$-P and native Jurkat T cells were treated with S-WGA-FITC of different concentrations (5, 10, 20, 40 and 80 μg/mL). (**a**) CLSM imaging (scale bar, 25 μm) and (**b**) FCM analysis of the binding level of S-WGA-FITC on cell surface. $c_{S\text{-}WGA\text{-}FITC}$, concentration of S-WGA-FITC. Data are representative of three independent experiments with similar results.

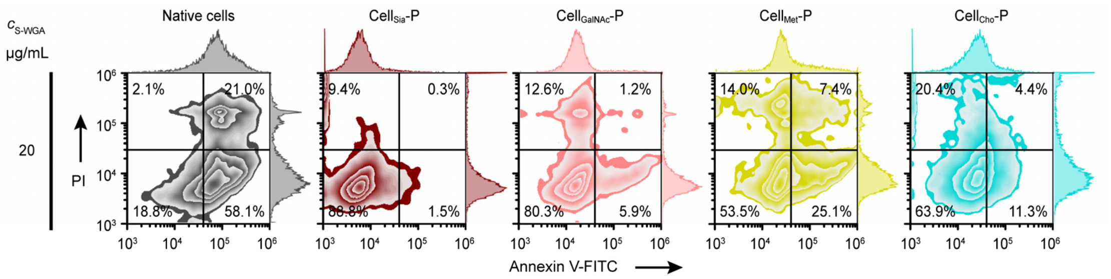

Supplementary Figure 32. Cell apoptosis analysis of cells upon S-WGA treatment. Zebra plots of native cells and different Cell-P after treatment with S-WGA of 20 μg/mL and Annexin V-FITC/PI staining. $c_{\text{S-WGA}}$, concentration of S-WGA. Data are representative of three independent experiments with similar results.

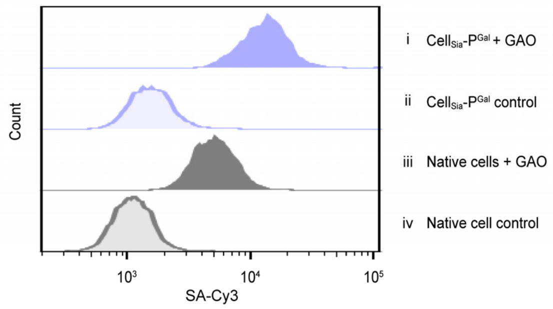

Supplementary Figure 33. Demonstration of the remodeling of artificial biomimetic glycocalyx. Cell$_{Sia}$-P^{Gal} and native cells were treated with GAO, followed by biotin hydrazide labeling, SA-Cy3 staining, and FCM analysis. Cell$_{Sia}$-P^{Gal} and native cells without GAO treatment were controls. Data are representative of three independent experiments with similar results.

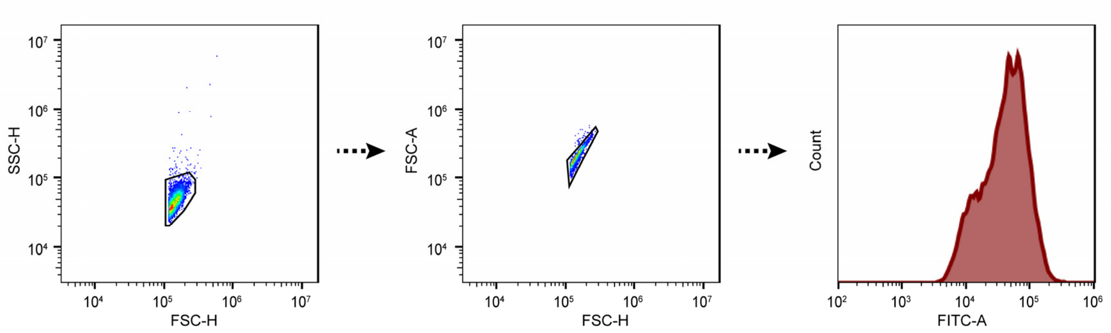

Supplementary Figure 34. Gating strategy for flow cytometric analysis (Supplementary Fig. 23a). Cells were gated by FSC/SSC to exclude cellular debris. Single cell population was gated with FSC-H/FSC-A. Depending on the study purpose, single cell population gating was not performed when cell aggregates were also analyzed.

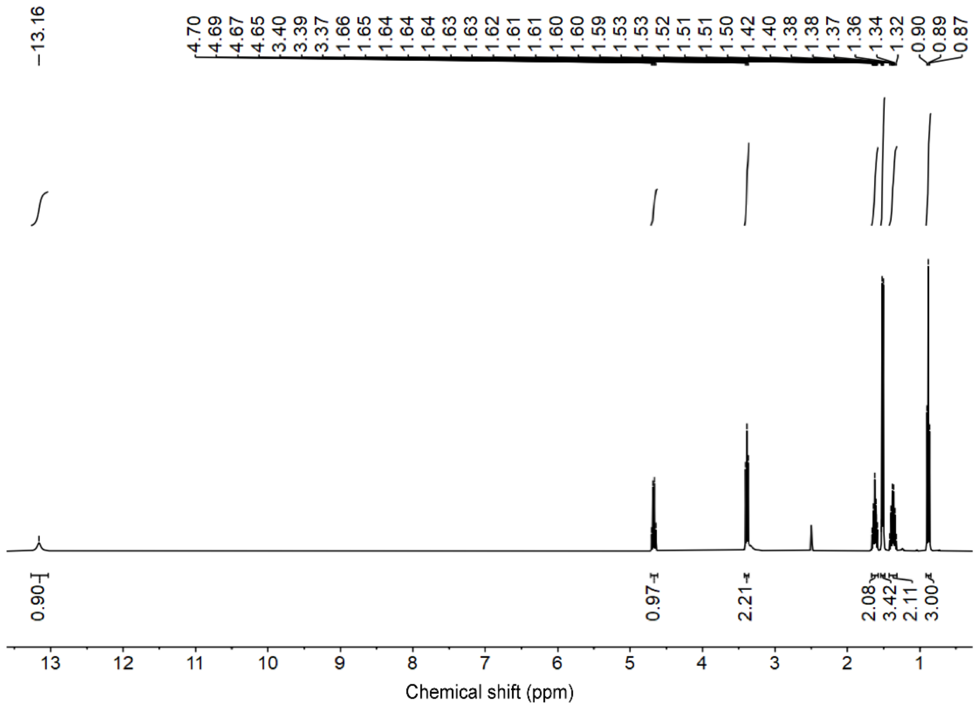

Supplementary Figure 35. ^{1}H NMR spectrum of BTPA.

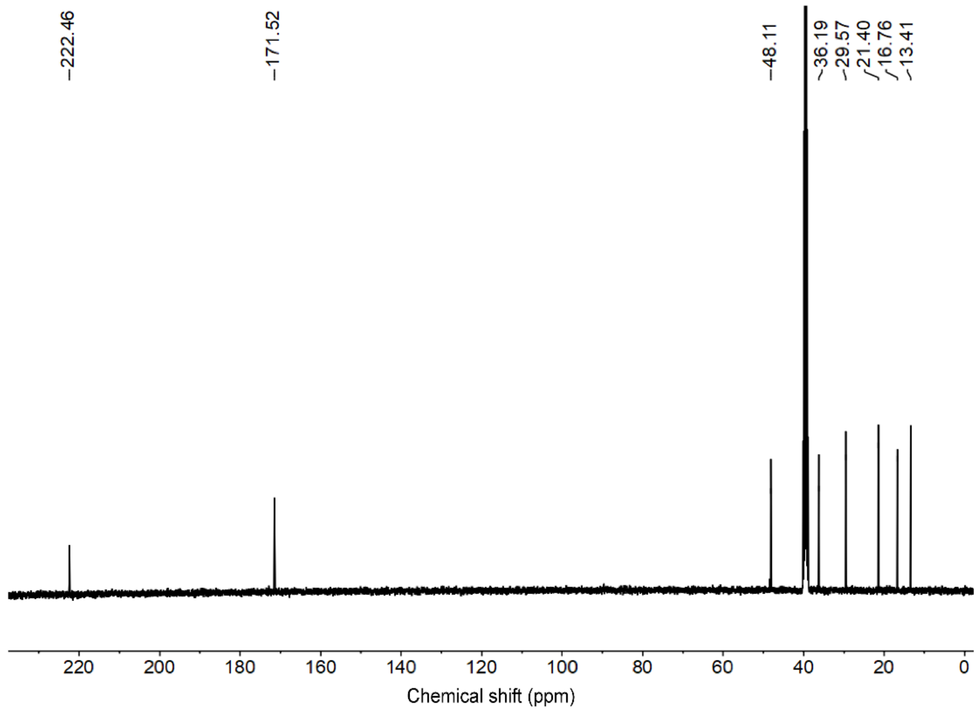

Supplementary Figure 36. ^{13}C NMR spectrum of BTPA.

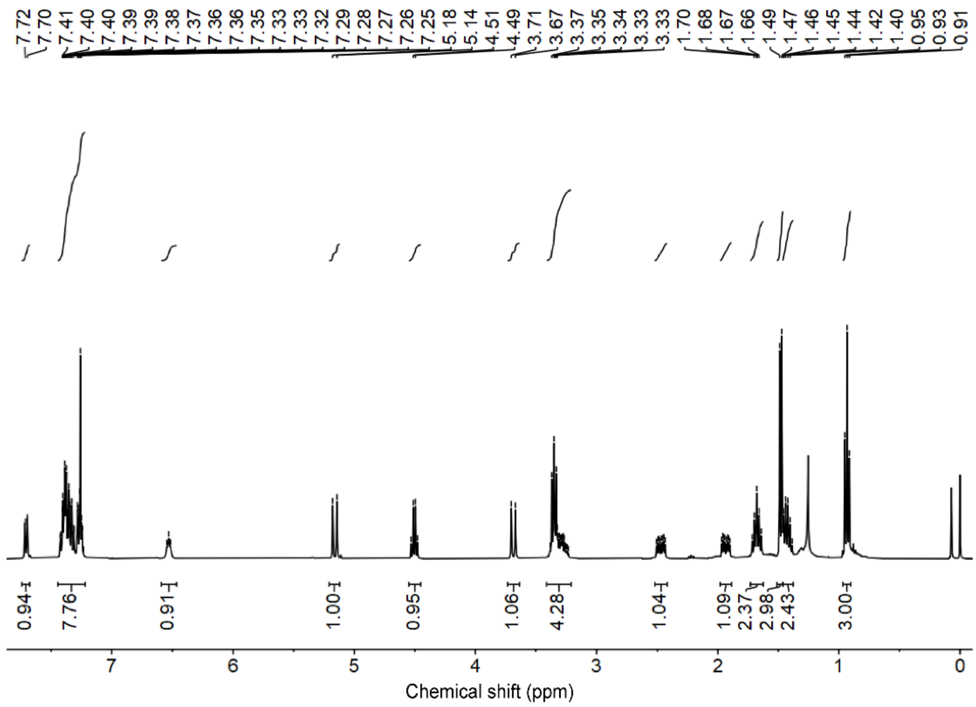

Supplementary Figure 37. ^{1}H NMR spectrum of DBCO-BTPA.

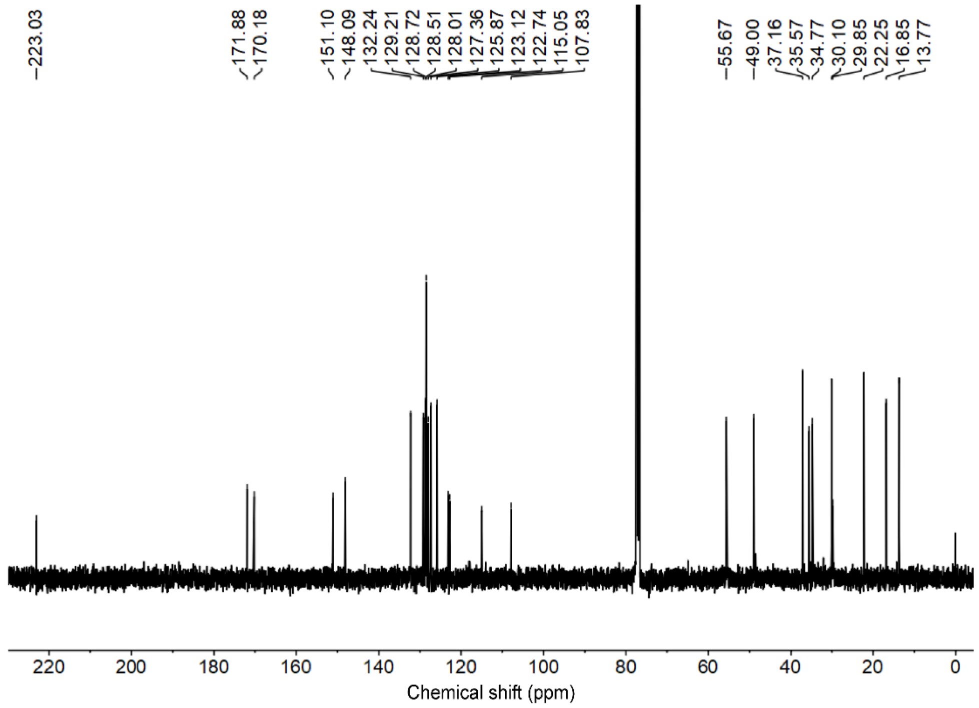

Supplementary Figure 38. ^{13}C NMR spectrum of DBCO-BTPA.

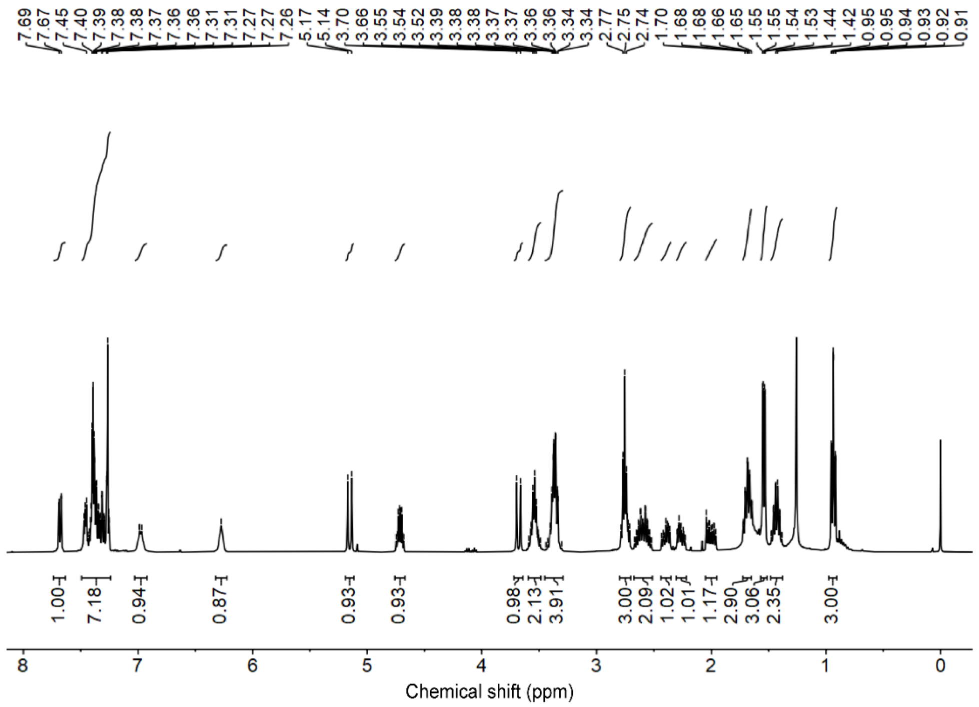

Supplementary Figure 39. ^{1}H NMR spectrum of DBCO-SS-BTPA.

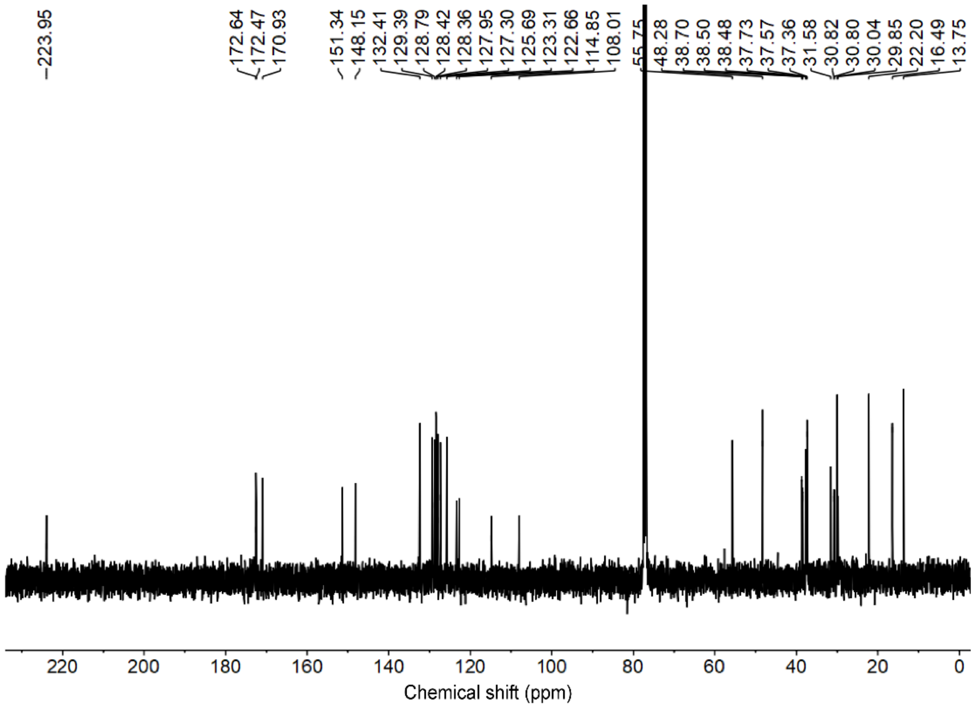

Supplementary Figure 40. ^{13}C NMR spectrum of DBCO-SS-BTPA.

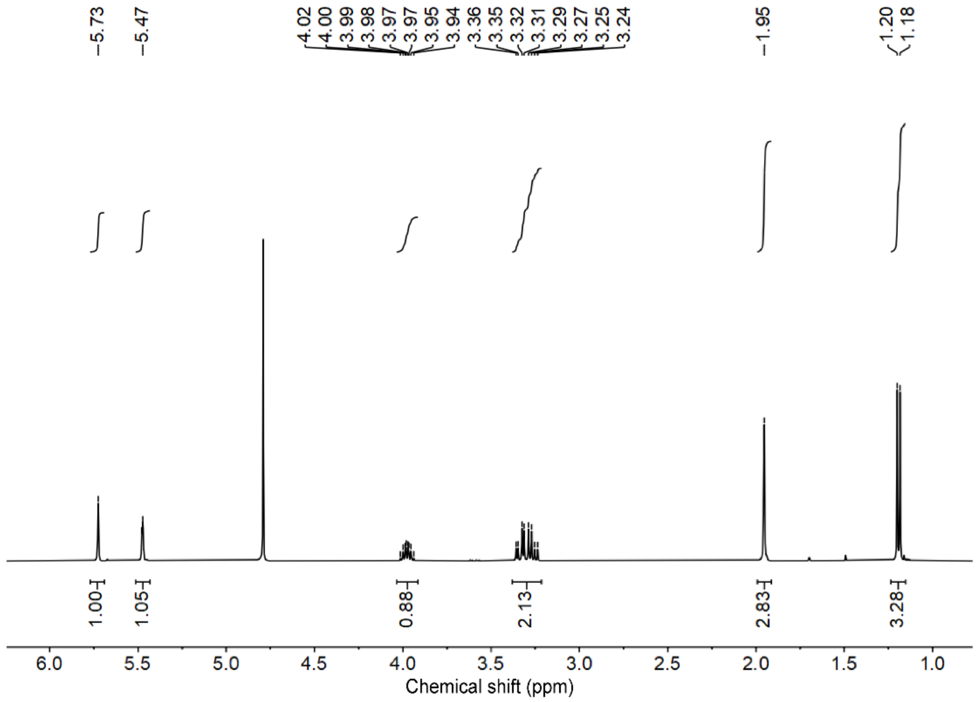

Supplementary Figure 41. ^{1}H NMR spectrum of HPMA.

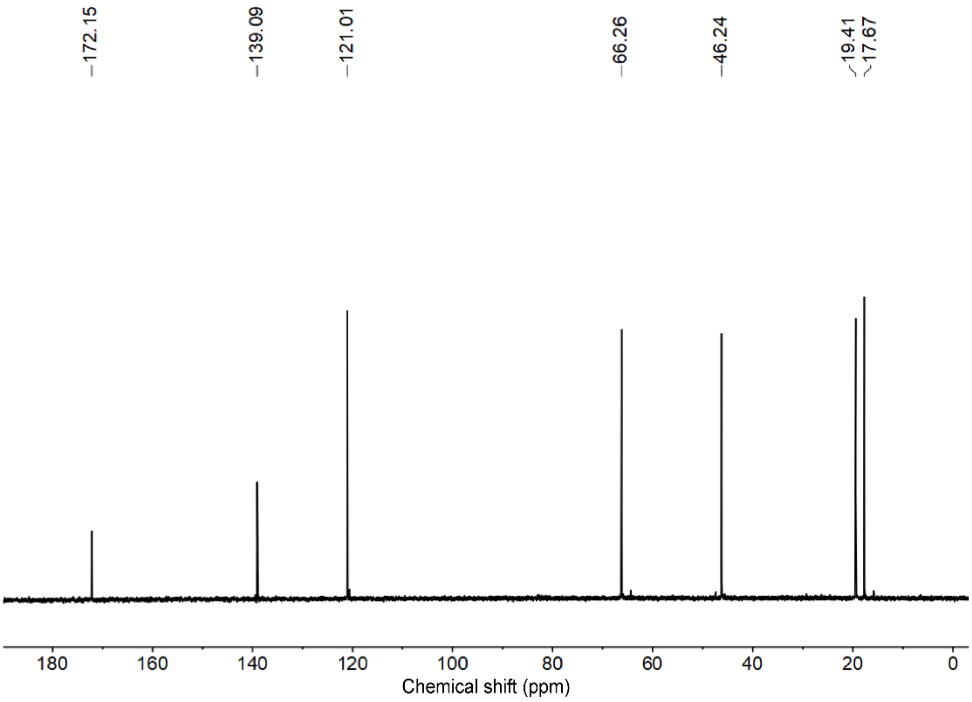

Supplementary Figure 42. ^{13}C NMR spectrum of HPMA.

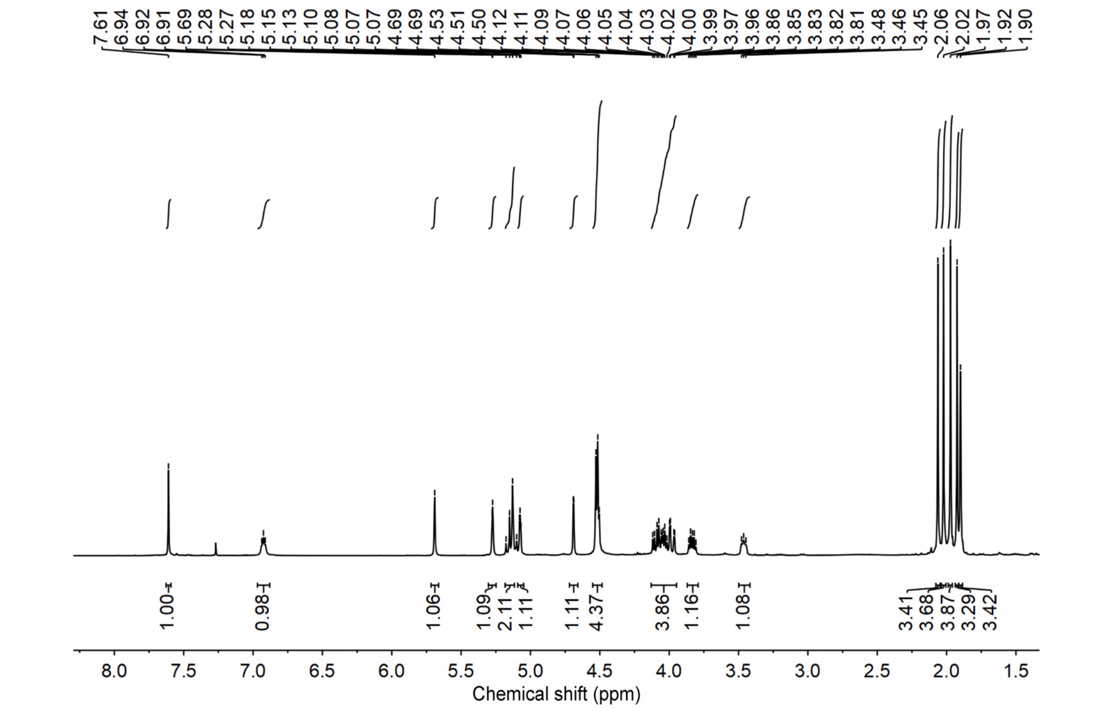

Supplementary Figure 43. ^{1}H NMR spectrum of **M3**.

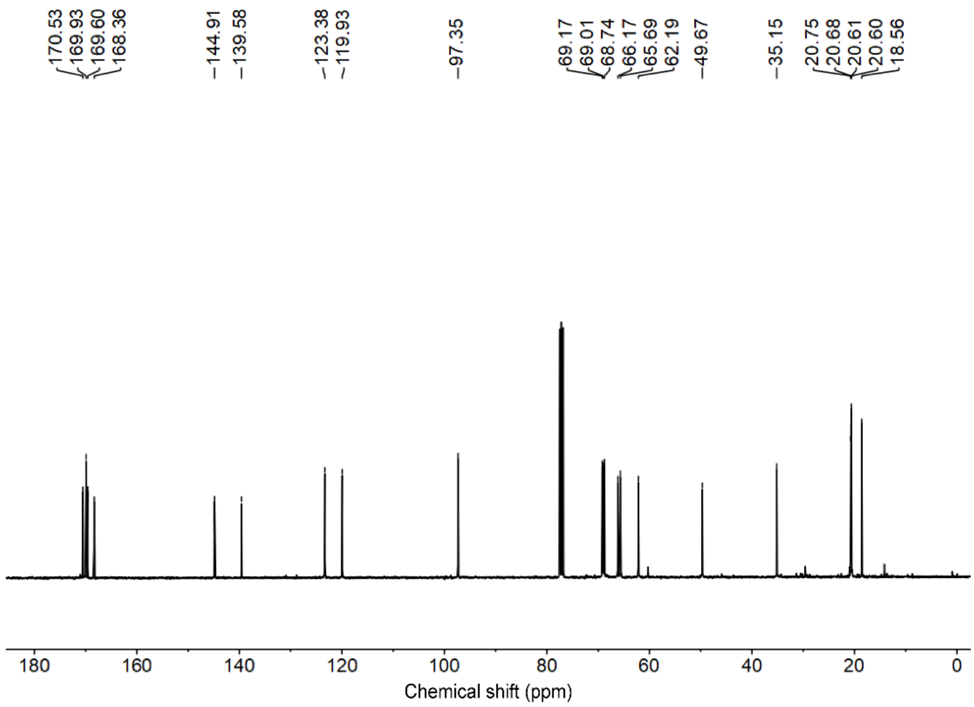

Supplementary Figure 44. ^{13}C NMR spectrum of **M3**.

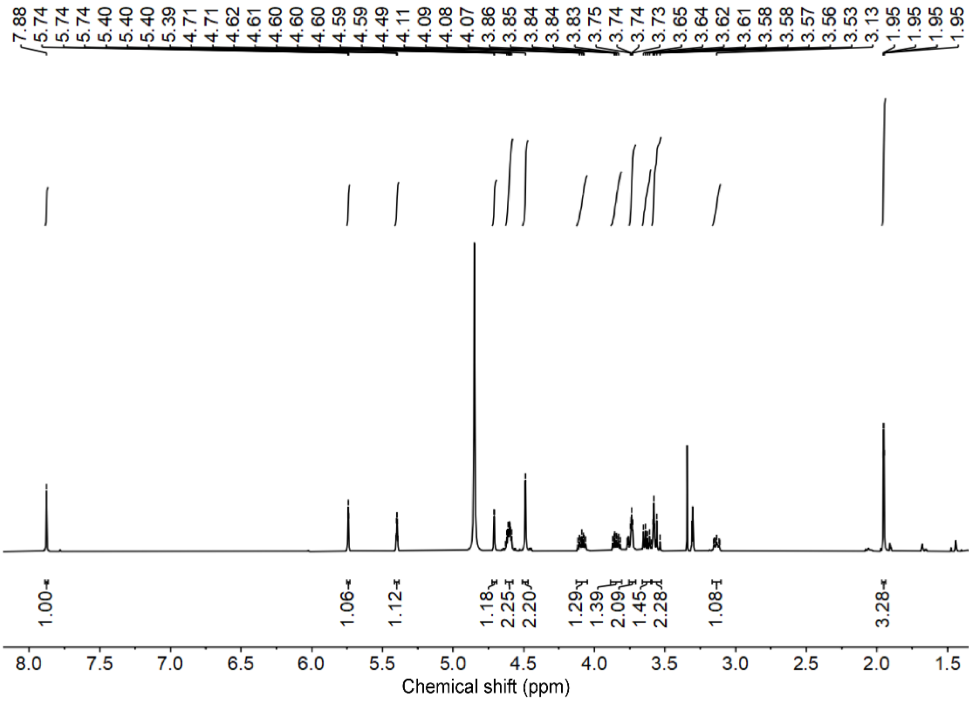

Supplementary Figure 45. ^{1}H NMR spectrum of MA-Man.

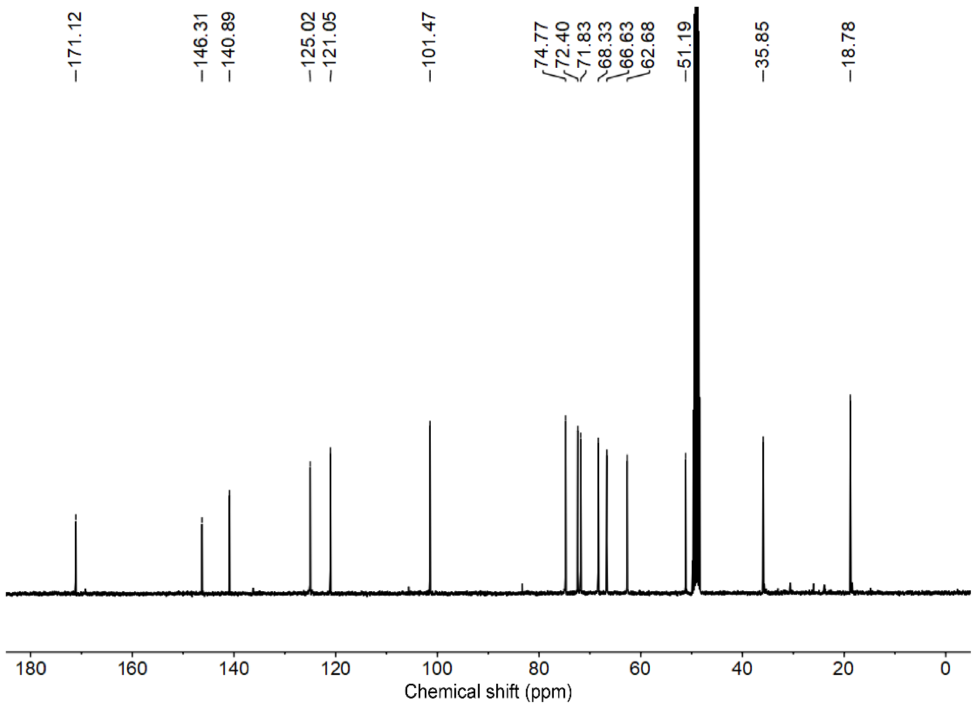

Supplementary Figure 46. ^{13}C NMR spectrum of MA-Man.

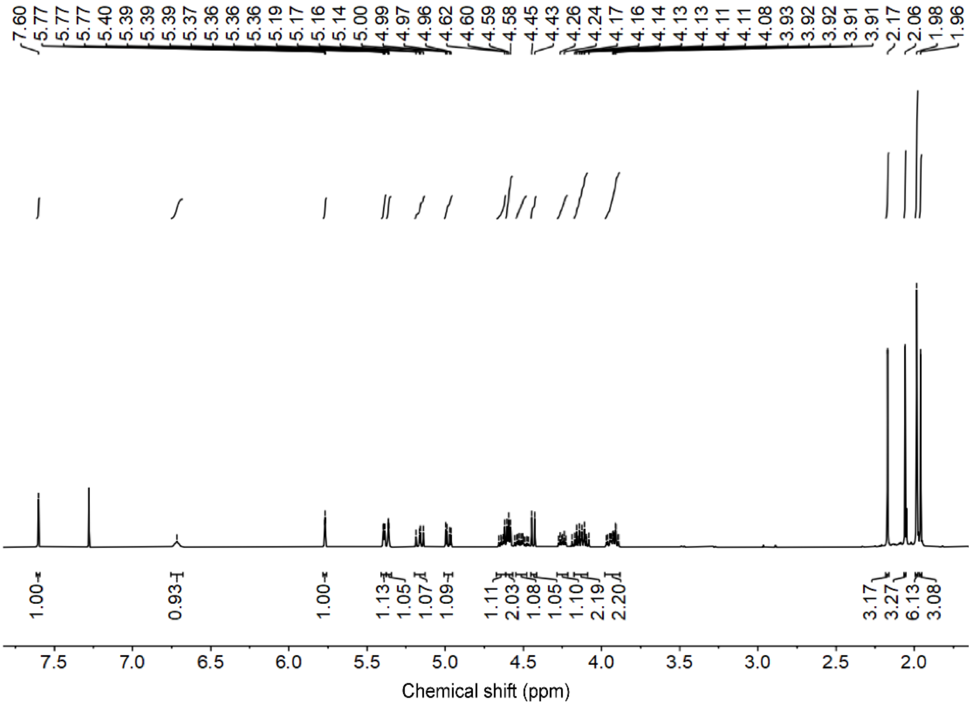

Supplementary Figure 47. ^{1}H NMR spectrum of **G3**.

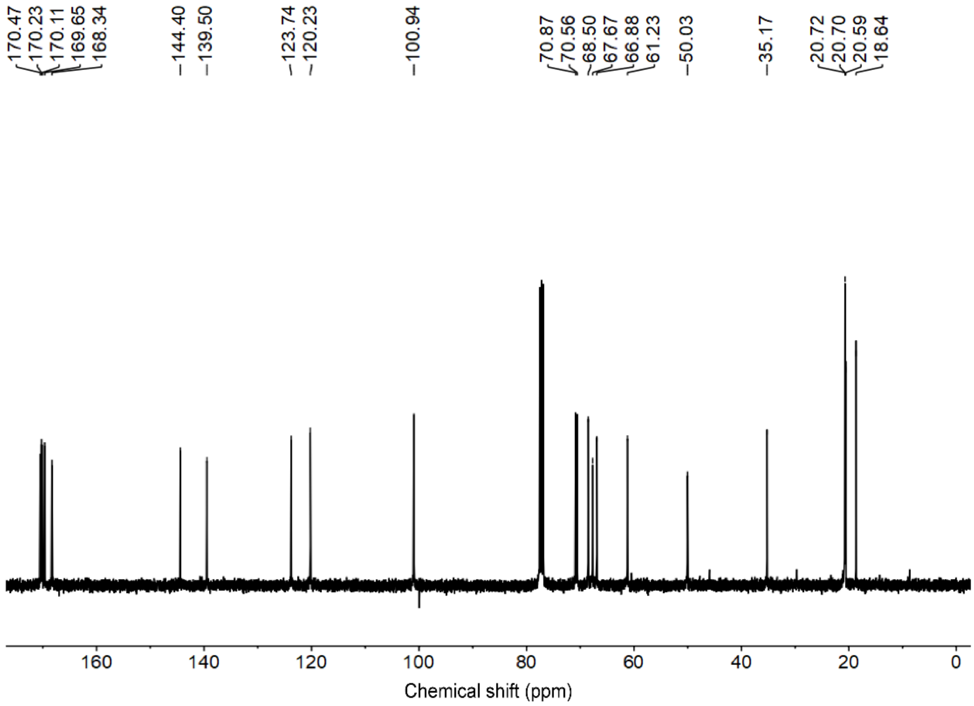

Supplementary Figure 48. ^{13}C NMR spectrum of **G3**.

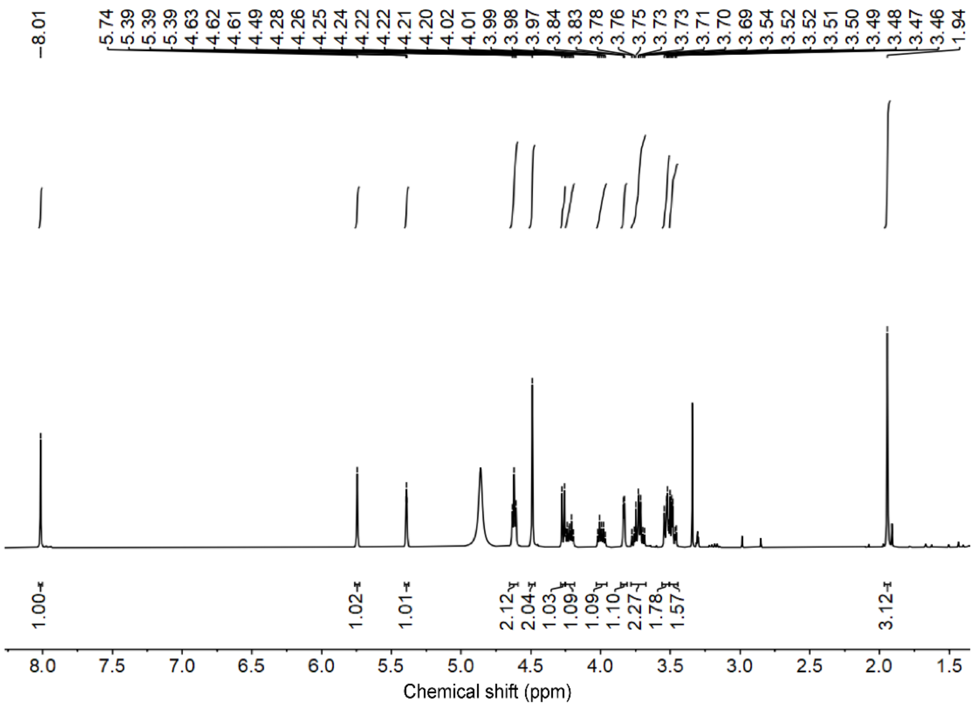

Supplementary Figure 49. ^{1}H NMR spectrum of MA-Gal.

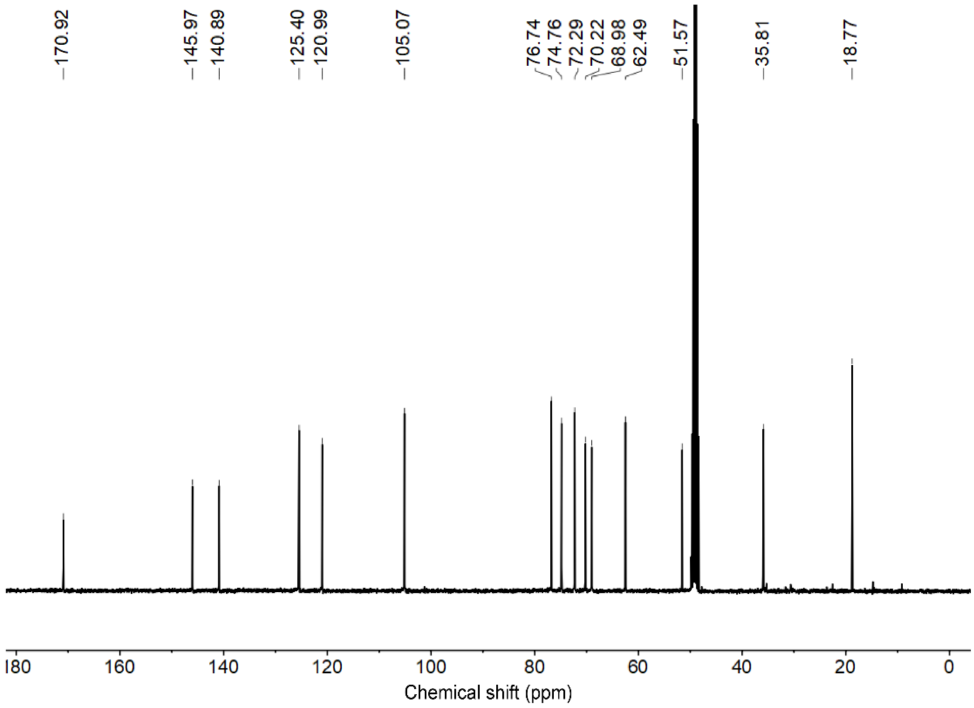

Supplementary Figure 50. ^{13}C NMR spectrum of MA-Gal.

3. Supplementary References

1. Ferguson, C. J. *et al.* Ab initio emulsion polymerization by RAFT-controlled self-assembly. *Macromolecules* **38**, 2191-2204 (2006).
2. Comegna, D., Gatto, A. D., Saviano, M. & Zaccaro, L. On-bead peptoid dimerization induced by incorporation of glycosylated bridging units in submonomer solid-phase approach to glycopeptoids. *Org. Lett.* **21**, 4454-4458 (2019).
